# Infant age inversely correlates with gut carriage of resistance genes, reflecting modifications in microbial carbohydrate metabolism during early life

**DOI:** 10.1002/imt2.169

**Published:** 2024-01-31

**Authors:** Xinming Xu, Qingying Feng, Tao Zhang, Yunlong Gao, Qu Cheng, Wanqiu Zhang, Qinglong Wu, Ke Xu, Yucan Li, Nhu Nguyen, Diana H. Taft, David A. Mills, Danielle G. Lemay, Weiyun Zhu, Shengyong Mao, Anyun Zhang, Kelin Xu, Jinxin Liu

**Affiliations:** ^1^ Laboratory of Gastrointestinal Microbiology, College of Animal Science & Technology Nanjing Agricultural University Nanjing China; ^2^ Jiangsu Key Laboratory of Gastrointestinal Nutrition and Animal Health, National Center for International Research on Animal Gut Nutrition Nanjing Agricultural University Nanjing China; ^3^ Department of Nutrition and Food Hygiene, School of Public Health, Institute of Nutrition Fudan University Shanghai China; ^4^ Biological Engineering Division Massachusetts Institute of Technology (MIT) Cambridge Massachusetts USA; ^5^ Department of Epidemiology and Biostatistics, School of Public Health, Tongji Medical College Huazhong University of Science and Technology Wuhan China; ^6^ Department of Pathology and Immunology Baylor College of Medicine Houston Texas USA; ^7^ Department of Statistics University of Chicago Chicago Illinois; ^8^ State Key Laboratory of Genetic Engineering, Human Phenome Institute Fudan University Shanghai China; ^9^ Department of Food Science and Technology University of California, Davis Davis California USA; ^10^ Department of Viticulture and Enology, Robert Mondavi Institute for Wine and Food Science University of California, Davis Davis California USA; ^11^ USDA ARS Western Human Nutrition Research Center Davis California USA; ^12^ Animal Disease Prevention and Food Safety Key Laboratory of Sichuan Province, Key Laboratory of Bio‐Resource and Eco‐Environment of Ministry of Education, College of Life Sciences Sichuan University Chengdu China; ^13^ Department of Biostatistics, Key Laboratory of Public Health Safety, NHC Key Laboratory of Health Technology Assessment, School of Public Health Fudan University Shanghai China

**Keywords:** age, antimicrobial resistance genes, carbohydrate metabolism, diet, infant gut resistome, metagenomics

## Abstract

The infant gut microbiome is increasingly recognized as a reservoir of antibiotic resistance genes, yet the assembly of gut resistome in infants and its influencing factors remain largely unknown. We characterized resistome in 4132 metagenomes from 963 infants in six countries and 4285 resistance genes were observed. The inherent resistome pattern of healthy infants (*N* = 272) could be distinguished by two stages: a multicompound resistance phase (Months 0–7) and a tetracycline‐mupirocin‐β‐lactam‐dominant phase (Months 8–14). Microbial taxonomy explained 40.7% of the gut resistome of healthy infants, with *Escherichia* (25.5%) harboring the most resistance genes. In a further analysis with all available infants (*N* = 963), we found age was the strongest influencer on the resistome and was negatively correlated with the overall resistance during the first 3 years (*p* < 0.001). Using a random‐forest approach, a set of 34 resistance genes could be used to predict age (*R* ^2^ = 68.0%). Leveraging microbial host inference analyses, we inferred the age‐dependent assembly of infant resistome was a result of shifts in the gut microbiome, primarily driven by changes in taxa that disproportionately harbor resistance genes across taxa (e.g., *Escherichia coli* more frequently harbored resistance genes than other taxa). We performed metagenomic functional profiling and metagenomic assembled genome analyses whose results indicate that the development of gut resistome was driven by changes in microbial carbohydrate metabolism, with an increasing need for carbohydrate‐active enzymes from *Bacteroidota* and a decreasing need for *Pseudomonadota* during infancy. Importantly, we observed increased acquired resistance genes over time, which was related to increased horizontal gene transfer in the developing infant gut microbiome. In summary, infant age was negatively correlated with antimicrobial resistance gene levels, reflecting a composition shift in the gut microbiome, likely driven by the changing need for microbial carbohydrate metabolism during early life.

## Highlights

- We first examined the natural assembly of resistome in healthy infants' guts and identified resistance genes that were significantly impacted by age.
- We inferred that the age‐dependent assembly of infant resistome was a result of the shift of the gut microbiome, which was due to resistance genes being disproportionally distributed across taxa.
- Age‐dependent assembly of infant resistome reflected the shift in the gut microbiome, and such modifications were often a result of dietary transitions during infancy.
- Maturation of gut resistome was driven by infants’ changing carbohydrate metabolism, which demonstrated an increasing need for carbohydrate‐active enzymes from *Bacteroidota* and decreasing involvements from *Pseudomonadota* during infancy.

## Graphical abstract

We infer that the maturation of infant resistome is not a random change but a programmed modification in the gut microbiome, which is due to resistance genes being disproportionally distributed across taxa. The decline of overall resistance is possibly due to the diminishment of *Pseudomonadota*, which is often equipped with antimicrobial resistance genes (ARGs), whereas the transition into table food creates a nutritional niche for *Bacteriodota* in the infant gut, which is a relatively small reservoir of resistance genes. The maturation of gut resistome is driven by infants’ changing carbohydrate metabolism, which demonstrated an increasing need for carbohydrate‐active enzymes from *Bacteroidota* and decreasing involvements from *Pseudomonadota* during infancy. AA, auxiliary activities; CAZy, carbohydrate‐active enzymes; GH, glycoside hydrolases; GT, glycosyltransferases; PL, polysaccharide lyases.

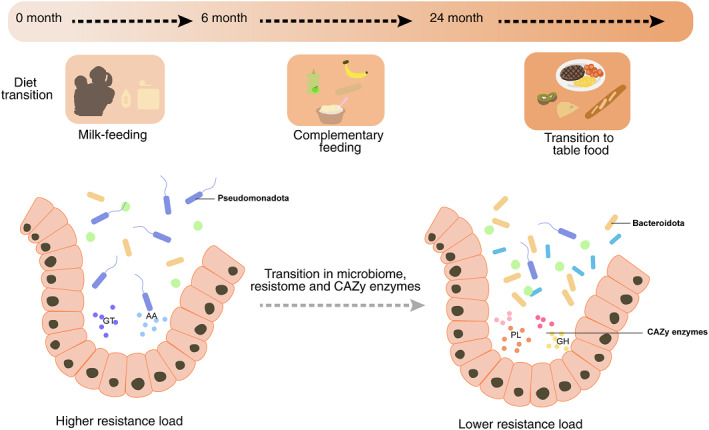

## INTRODUCTION

The assembly of infant gut microbiota occurs soon after birth, in close concert with host immune system development, host metabolism, and host intestinal homeostasis [1, 2, 3, 4]. Colonization occurs from multiple sources [5], and the developing infant gut microbiota can be divided into three phases: a developmental phase (Months 3–14), a transitional phase (Months 15–30), and a stable phase (≥31 months) [6]. Studies have demonstrated that host genetics, prenatal intrauterine environment, and the mode of delivery are associated with the composition of infant gut microbiomes at birth [7, 8]. Multiple factors, including geography [9], antibiotic treatment, the method of feeding [10], age at weaning, and environmental exposures, will further influence microbiome maturation [4]. The developmental and transitional phases of infant gut microbiota development are considered vital windows when microbe‐based interventions to reduce the risk of diseases and improve overall host health may be possible [11].

Coincident with the development of infant microbiota, the gut resistome (i.e., the collection of all resistance genes in a biome) is assembled. The infant gut microbiota are recognized as reservoirs of antimicrobial resistance genes (ARGs), and one recent study found that 40% of detected ARGs found in the infant gut conferred resistance to multiple antibiotics, including resistance to drugs to which infants had not been exposed [12]. Globally, ~214,000 cases of neonatal sepsis‐related death are attributed to ARG‐carrying pathogens [13]. Previous studies have demonstrated that the infant gut microbiota matures in an age‐dependent manner [14, 15], whether this is also true for the resistome and how microbiota influencing factors might affect the trajectory of resistome remains understudied. Therefore, understanding the natural assembly of the gut resistome and which factors influence the resistome is a priority for public health.

We aim to study the infant gut resistome's natural assembly, including identifying factors that influence the trajectory of resistome development using publically available metagenomes from infants and toddlers. Our findings present a landscape of the infant gut resistome, which will lay the foundation for studies in developing strategies to mitigate the prevalence of antimicrobial resistance.

## RESULTS

### Overview of multicohort worldwide infant gut metagenomes

**Figure 1:**
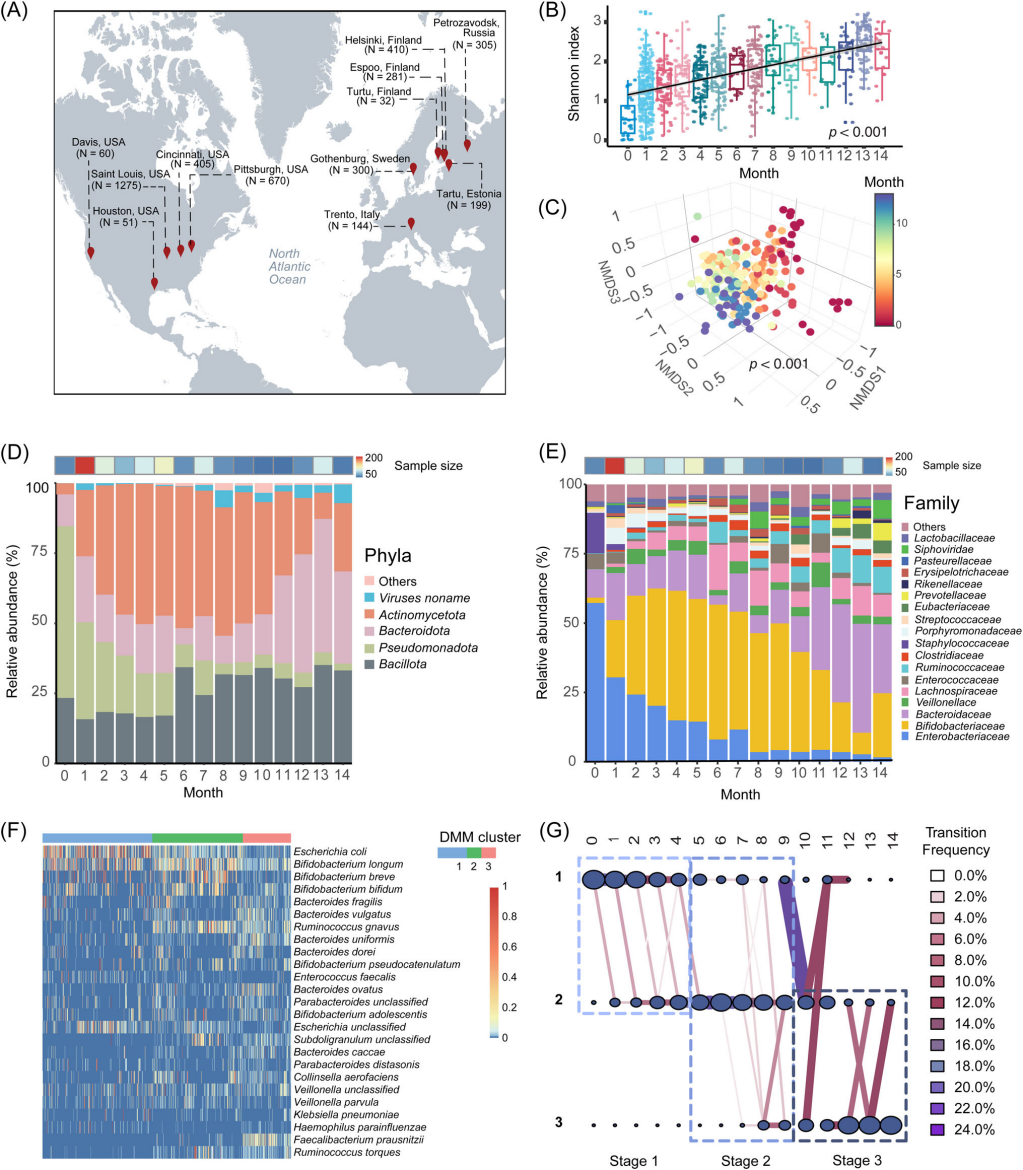
Sampling geographical distribution and the assembly of healthy infant gut microbiota during the first 14 months of their life. (A) Geographical distribution of 4132 infant‐related metagenomes from 963 infants in Estonia (*N* = 71), Finland (*N* = 170), Italy (*N* = 30), Russia (*N* = 70), Sweden (*N* = 100), and United States (*N* = 522). (B) Box plot depicting the α‐diversity of healthy infant gut microbiome as measured by Shannon diversity (*N* = 858). Dots represent individual samples and Shannon index values were presented as the median (central horizontal line); the lower and upper hinges correspond to the 25th and 75th percentiles, respectively. A generalized linear mix regression model was represented by the regression line with a 95% confidence interval in gray shading. (C) Nonmetric multidimensional scaling (NMDS) of healthy infant fecal samples based on a Bray–Curtis calculation (*k* = 4, stress = 0.1). Subsampling to obtain an equal sample size (*N*  =  20) at each month (Months 10, 11, and 14 were deleted due to sampling size < 20) was completed before performing the ordination analysis and permutational multivariate analysis of variance (PERMANOVA). The centroid of each ellipse represents the group mean, and the shape was defined by the covariance within each group. Because of limited inter‐sample variation, samples belonging to months 8–11 and 12–14 were labeled as connected groups. (D, E) Stacked bar plots depicting the relative abundance of microbes as measured at the phyla and family level, respectively. Microbial taxa quantified with an average relative abundance <1% were aggregated into “Others”. (F) Heatmap indicating the relative abundance of the top 25 dominant bacterial species as classified in three Dirichlet multinomial mixtures (DMM) clusters. DMM model was constructed by all microbes at the species level. From top to bottom, microbes were listed in an order with a decreased contribution to the DMM model. (G) A transition model showing the progression of bacterial species across DMM clusters over time. Nodes represented the sample size per month in each cluster. The color and thickness of edges represented the transition frequency, as measured by the proportion of the microbes that transitioned from one node to the next.

To assemble a cohort of global infant gut metagenomes, we retrieved 4132 infant and toddler (under age 3 years)‐related metagenomes from 963 infants enrolled in 19 different cohorts, in 17 independent studies between 2015 and 2020 (Figure 1A and Table S1). The sequencing platforms used by the studies were Illumina HiSeq. 2000 (*N* = 7), Illumina HiSeq. 2500 (*N* = 7), Illumina NextSeq. 500 (*N* = 4), and Illumina HiSeq. 4000 (*N* = 2). An additional three studies generated sequences via multiple platforms. We applied a unified quality control step to handle the study‐dependent variance in sequencing quality, and the overall quality was significantly improved through this data pre‐processing (Figure S1).

Of the 963 infants included in our assembled cohort, 38.6% (*N* = 372) were female, 38.1% (*N* = 367) were male, and 23.3% (*N* = 224) were not recorded with a sex marker. Then, 36.0% of these infants (*N* = 346) were preterm and 63.8% (*N* = 614) were full‐term. Next, 64.7% (*N* = 623) were vaginally delivered and 34.6% (*N* = 333) were delivered by cesarean section (Table S1). In addition, 4.8% (*N* = 46) of included infants experienced necrotizing enterocolitis (NEC) (all of them were preterm) and 29.1% (*N* = 280) had a recorded episode of antibiotic treatment. Samples were collected from a wide array of geographical locations (Estonia, Finland, Italy, Russia, Sweden, and the United States) (Figure 1A), representing a >3‐year long (i.e., day 0 to day 1162) development of infant gut microbiome and resistome. The assembled metagenomes and associated clinical traits of infants enabled the investigation of the trajectory of gut resistome development during early life and the identification of factors with the greatest influence on resistome development.

### Natural assembly of healthy infant gut microbiota during early life

Full‐term infants who were vaginally delivered without any recorded antibiotic treatment were categorized as “healthy” in our analysis. Consequently, 858 consecutive stool metagenomes from 272 infants during the first 14 months of life were used to capture the natural assembly of healthy infant gut microbiota.

We observed a significant increase in α‐diversity as measured by the Shannon index over time, indicative of the actively assembling gut microbiome (linear mixed model [LMM], *p* < 0.001, Figure 1B). There were distinct fecal metagenome structures based on infant age in months at the time of collection (permutational multivariate analysis of variance [PERMANOVA], *p* < 0.001, Figure 1C). *Actinomycetota*, *Bacteroidota*, *Bacillota*, and *Pseudomonadota* dominated the developing gut microbiome, representing ~99.82% of all profiled microbes (Figure 1D). The relative abundance of *Enterobacteriaceae* decreased drastically over 14 months, whereas *Bifidobacteriaceae* and *Bacteroidaceae* increased (Figure 1E).

The developing infant gut microbiome was classified into three enterotype clusters by a Dirichlet multinomial mixtures (DMM) model, with Cluster 1 dominated by bacterial genus *Escherichia*; Cluster 2 dominated by *Bifidobacterium*; and Cluster 3 dominated by *Bacteroides* (Figure 1F). Our DMM modeling observed a salient enterotype transition over time with three distinct developmental stages (Figure 1G). Microbes observed in fecal metagenomes from Month 0 to 4, Month 5 to 9, and Month 10 to 14 were mainly classified in Cluster 1, Cluster 2, and Cluster 3, respectively (Figure 1G). Consequently, our assembled cohort of metagenomes represents a typical development of infants’ gut microbiome during early life consistent with earlier studies [6, 10].

### The typical succession of fecal resistome during the first 14 months of life in healthy infants

**Figure 2:**
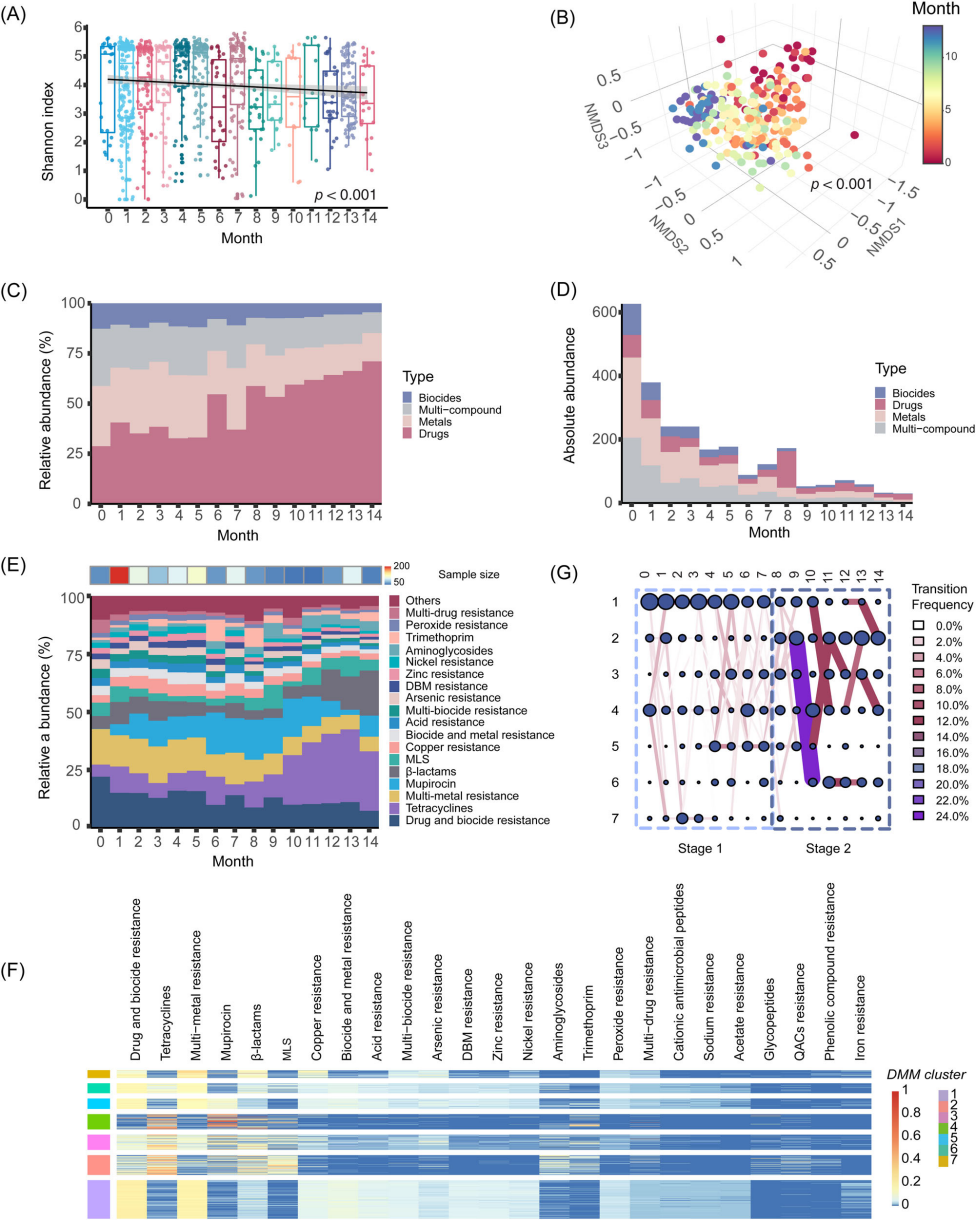
The assembly of gut resistome in healthy infants during early life. (A) box plot depicting the α‐diversity of infant gut resistome as measured by Shannon index (*N* = 858). A generalized linear mix regression model was represented by the regression line with a 95% confidence interval in gray shading. (B) Nonmetric multidimensional scaling (NMDS) of healthy infant gut resistome based on a Bray–Curtis dissimilarity calculation at the individual resistance gene level (*k* = 4, stress = 0.1). Subsampling to obtain an equal sample size (*N*  =  20) at each month (months 10, 11, and 14 were deleted due to sampling size < 20) was completed before performing the ordination analysis and permutational multivariate analysis of variance (PERMANOVA). (C) Absolute abundance of resistance genes as measured by resistance reads per kilobase per genome equivalent (RPKG) at the type level. (D, E) Stacked bar plot depicting the relative abundance of resistance groups as measured at the type and class level of resistome, respectively. Resistance groups quantified with an average relative abundance <1% were aggregated into “Others.” (F) Heatmap depicting the relative abundance of the 25 most descriptive antimicrobial classes across Dirichlet multinomial mixture (DMM) clusters. From left to right, resistome classes were listed in an order with a decreased contribution to the DMM model. DMM model was constructed by all resistome at the class level. (G) A transition model showing the progression of resistome across DMM clusters over time. Nodes represented the sample size per month in each cluster. The color and thickness of edges represented the transition frequency, as measured by the proportion of resistances from antimicrobial classes that transitioned from one node to the next.

We next examined the fecal resistome in healthy infants. During the first 14 months of life, 2732 resistance genes were observed in the gut microbiome. The gut resistome α‐diversity was inversely associated with infant age (LMM, *p* < 0.005, Figure 2A). Consistent with the gradually changing microbial community, we observed gradual changes in the infant resistome with increasing age (PERMANOVA, *p* < 0.001, Figure 2B). Of the resistance types included in the MEGARes database, the drug resistance type was the most frequently observed, followed by metal, multi‐compound, and biocide resistance types (Figure 2C). At birth, the relative abundance of sequences conferring resistance to each of the MEGARes types (biocides, drugs, metals, and multicompound) was comparable; however, the level of drug type resistance gradually increased to 91.3% of all resistance sequences (median; interquartile range [IQR], 55.6%) at Month 14 (Figure 2C). The absolute abundance of resistance genes, as quantified by resistance‐related reads per kilobase per genome equivalent (RPKG), decreased over time (LMM, *p* < 0.05, Figure 2D). Specifically, the meconium microbiome harbored the highest abundance of resistance (>600 RPKG), indicating the presence of a natural reservoir of resistance for infants at birth (Figure 2D). Overall, resistance to tetracyclines (14.7%) and drug‐biocide (12.8%) were the most common antimicrobial resistance phenotypes in the developing infant guts within the first 14 months, followed by resistance to multi‐metal (11.0%), mupirocin (10.6%), β‐lactams (7.9%), macrolides–lincosamides–streptogramines (4.9%), and copper (4.0%) (Figure 2E).

The DMM model identified the succession of infant gut resistome characterized by seven resistance clusters on resistance at the class level (Figure 2F,G). Over 14 months, the healthy infant gut resistome exhibited two distinct developmental stages: the multicompound resistance stage (Months 0–7), showing a dominant level of drug‐biocide resistance (15.1% ± 3.4%) (mean ± SD) and multimetal resistance (14.0% ± 1.9%); and the drug resistance dominant stage (Months 8–14), during which tetracycline (22.3% ± 8.7%), mupirocin (11.4% ± 5.6%), and β‐lactam (9.4% ± 6.3%) resistances were prevalent (Figure 2G). Importantly, clinically relevant antimicrobial resistance genotypes (e.g., β‐lactam antibiotics) were conserved and remained dominant in infant gut microbes, posing a threat to the potential treatment of antibiotics, including penicillin and amoxicillin [16].

### *Escherichia* harbored the most resistance genes in healthy infants

**Figure 3:**
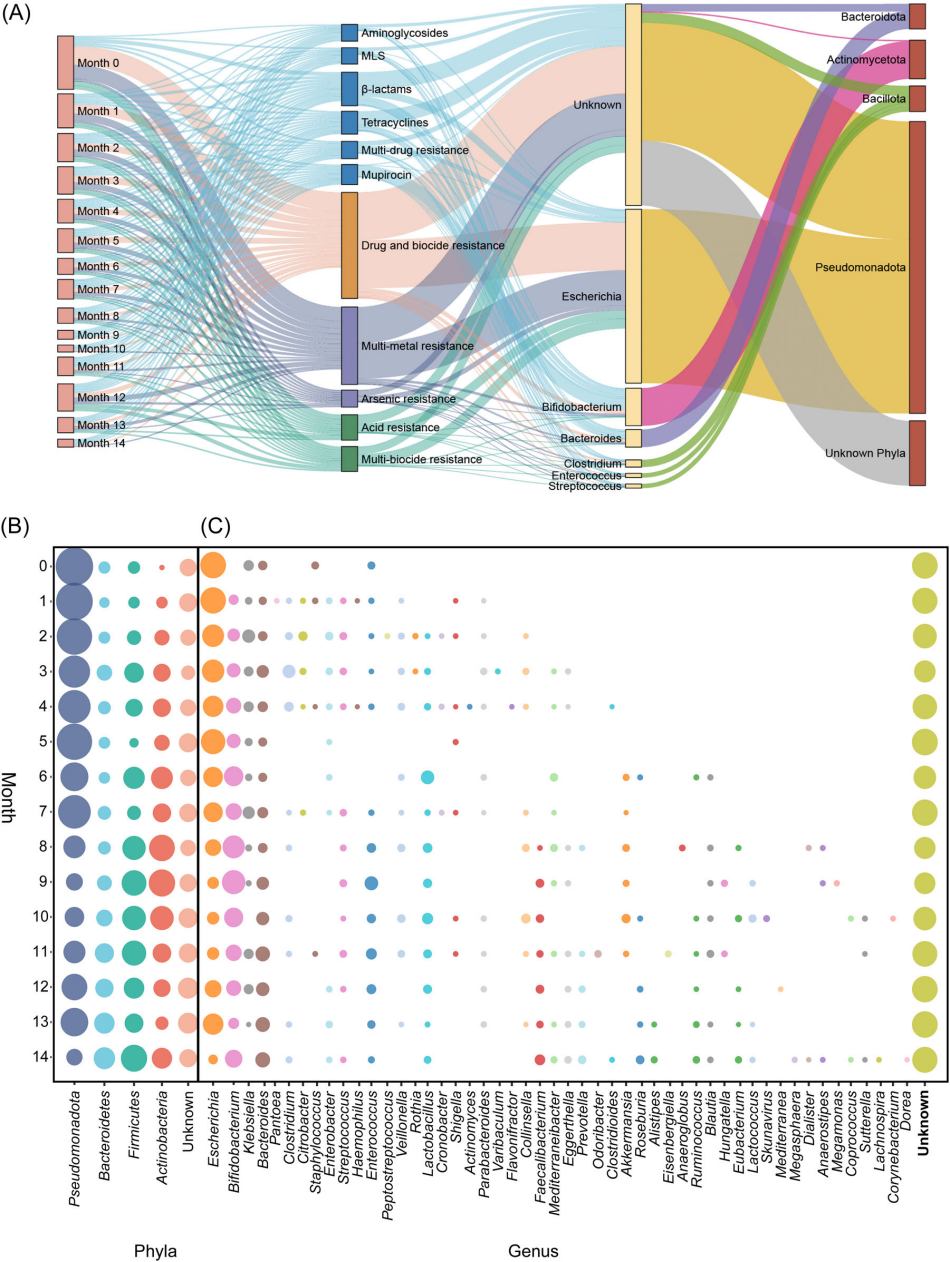
Microbial hosts of healthy infant gut resistome. (A) Sankey diagram connecting resistance genes from months (the first column) at the antimicrobial compound class level (the second column) to the predicted bacterial hosts at the genus (the third column) and phyla level (the fourth column). Phyla and Genus were respectively limited within the Top 4 phyla and Top 4 genera carrying the most resistance; otherwise, will be aggregated to other phyla or other genera. (B, C) Bubble plots depicting the dynamic change of bacterial hosts of resistance genes at the phyla and genus level over time. For better visualization, phyla and genus appeared in <25%, <20% of sample numbers (*N* = 851), and were aggregated into other phyla and other genera, including unknown.

The increasing complexity of the infant gut microbiome is accompanied by a decline in the absolute abundance of resistance genes, suggesting an uneven distribution of these resistance genes across microbial taxa. Our LMM analysis, by incorporating the microbiome distance matrix into the final model, demonstrated that 36.1% of the variation in aggregated resistance (as measured by the sum of resistance to drugs, metals, biocides, and multicompounds) could be explained by bacterial genera. By assigning a microbial taxon to the resistance gene‐containing contigs, we found that *Pseudomonadota* was the dominant resistome‐related phyla, which harbored 3720 unique resistance genes (3809 in all), representing 46.9% of the infant gut resistome overall abundance (Figure 3A and Figure S2).

Approximately 75.4% of resistance genes (*N* = 2040) were from *Pseudomonadota* at birth and the contribution of *Pseudomonadota* to the resistome gradually decreased to 13.9% of resistance genes at month 14, comparable to levels of *Actinomycetota* (0.9% at birth, 20.9% at Month 14), *Bacillota* (5.0% at birth, 30.5% at Month 14), and *Bacteroidetes* (10.4% at birth, 22.1% at Month 14) (Figure 3B). Microbes from 1273 genera were predicted to possess resistance traits in healthy infants’ gut, with *Escherichia* (25.5%) and *Bifidobacterium* (12.8%) harboring the most resistance genes, followed by *Bacteroides* and *Klebsiella* (Figure 3C). The resistome from *Escherichia* was dominated by drug‐biocide resistance (24.1%) and multimetal resistance (18.2%), whereas *Bifidobacterium* frequently harbored resistance to mupirocin (25.2%), β‐lactams (13.4%), and drug‐biocide (13.3%) (Figure 3A and Figure S3).

### The developing gut resistome is age‐dependent during infancy

**Figure 4:**
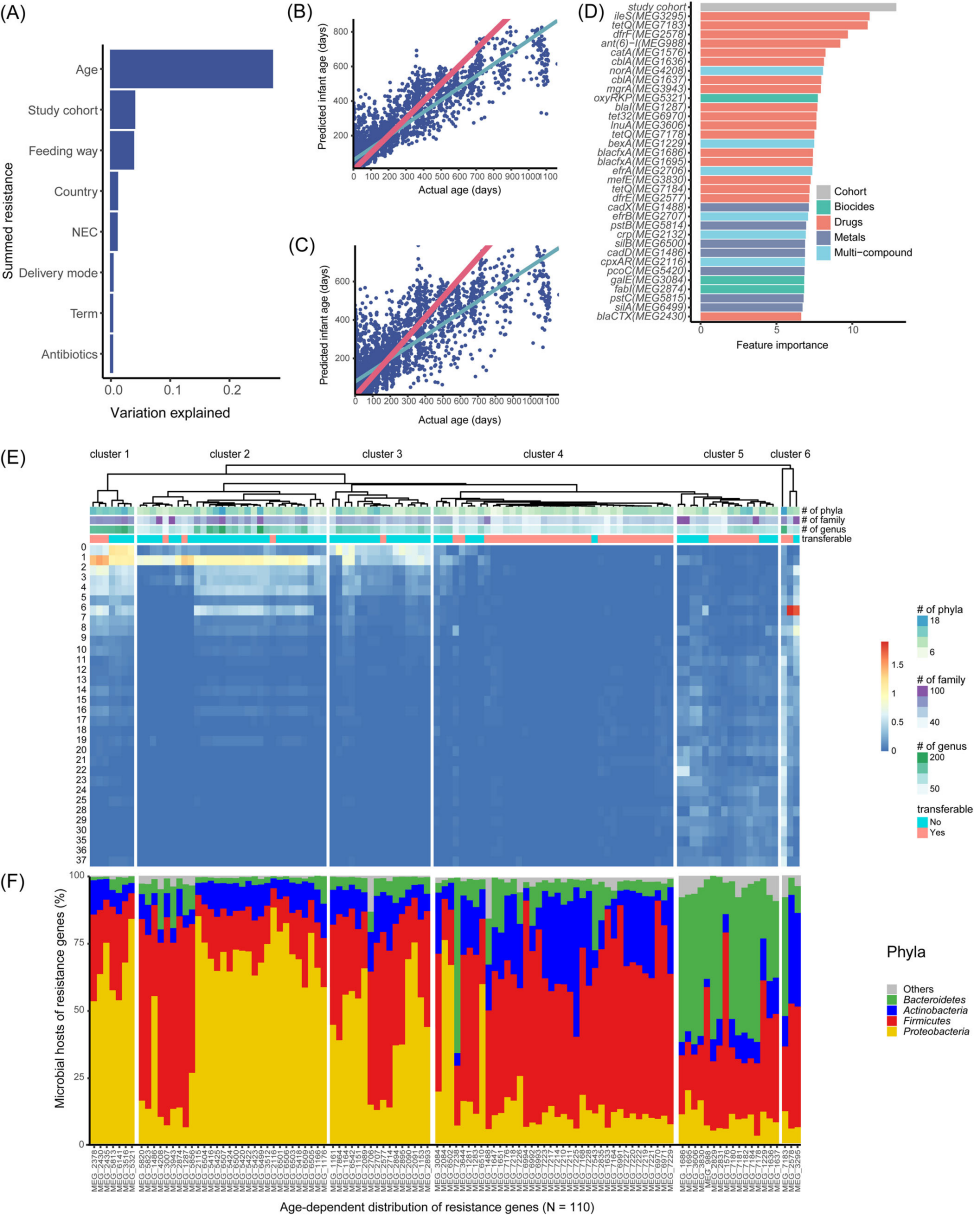
Factors influencing the assembly of the infant gut resistome and age‐discriminatory resistance genes. (A) Variation explained by the clinical variables. (B, C) The predicted infant age (resistome age) versus actual infant age in the (B) test set and (C) training set in the random forest (RF) model. The full data set was split into a training data set (*N* for infant host): test data set (*N* for infant host) = 7:3. The pink line represents the time when the actual age (Days) equals the predicted infant age (Days). The petrol line represents the fitted line based on a linear model [*R* ^2^ = 68.0%]. (D) Ranked lists of the Top 34 MEGIDs (antimicrobial resistance gene IDs in MEGARes, https://www.meglab.org/) in their group format (resistome genes) whose absolute abundance values when permuted have a larger marginal increase in MSE (mean squared error) besides the study cohort. They are consistent with the “infant age discriminatory importance”. The plot below shows the cross‐validation error versus the number of MEGIDs. Ten‐fold cross‐validation indicates that 35 variables are sufficient for RF predictions of the infant age day based on resistance composition. The inset details vertex. Data are mean ± SEM computed over 100 iterations. (E) Heatmap depicting the abundance of resistance genes, which were significantly associated with age over 37 months (*N* = 103). The abundance values were log‐transformed (log [value + 1, 10]) for better visualization. The numbers of distinct microbial hosts of individual genes were calculated at the level of bacterial genus, family, and phyla. The transfer potential of these resistance genes was characterized by manually referring to published experimental data. (F) Stacked bar plots demonstrating the microbial hosts of individual resistance genes at the level of phyla. NEC, necrotizing enterocolitis.

Next, we extended our analysis from healthy infants to all metagenomic samples to explore the factors influencing the infant gut resistome. When including all samples, we identified 4285 resistance genes. A LMM indicated that clinical variables and study cohort (fixed effects) explained 37.9% of the variation in overall resistance. The study cohort, age, method of feeding, and NEC were significantly associated with the overall resistance (Figure S4). Age was negatively correlated with overall resistance, explaining 27.5% of the variance in overall resistance abundance (Figure 4A).

We further applied a random forest (RF) model to identify time‐specific signatures in the resistome. The developing gut resistome was linearly related to infant biological age; this suggests a deterministic transition of resistance over time (Figure 4B,C). RF, with a cross‐validation analysis (*R* ^2^ = 68.0%), identified the 34 most predictive resistance genes most predictive of age, of which 52.9% conferred resistance to drugs, 17.6% conferred multicompound resistance, 8.8% conferred biocide resistance, and 20.6% conferred metal resistance (Figure 4D and Table S2). A mupirocin resistance gene, *ileS*, was ranked as the most important variable differentiating infant age (Figure 4D and Figure S5). Multiple *tetQ* resistance genes (i.e., MEG7183, MEG7178, and MEG7184) exhibited a gradual increase in absolute abundance over time and were strong indicators of infant age (Figure S5). Resistance from class A β‐lactams (i.e., MEG1636, MEG1637) showed a similar pattern (Figure S5). Two arsenic resistance genes, *pstB*, and *pstC* (i.e., MEG5815), decreased in absolute abundance with infant age. This decrease was also observed with multi‐biocide resistance (MEG5321) and multicompound (MEG2132) genes (Figure S5).

To decipher the underlying mechanism of the age‐dependent assembly of infant gut resistome, we attempted to identify the resistance genes that exhibited significant correlations with age in terms of their abundance. Overall, 110 genes were grouped into six clusters with two distinct dynamic patterns: the abundance of resistance genes belonging to Clusters 1–3 (*N* = 53) was relatively high at the beginning and then decreased over time, while genes classified within Clusters 4–6 (*N* = 57) gradually increased during infancy (Figure 4E). Genes in Clusters 1–3 originated from more taxa than genes in Clusters 4–6. In Clusters 1–3, genes originated from an average of 11.3 phyla (±2.6), 67.8 families (±15.7), and 136.0 genera (±41.5). In Clusters 4–6, genes originated from an average of 9.1 phyla (±2.2), 47.5 families (±12.0), and 93.9 genera (±27.4). Genes from Clusters 4–6 (ratio of genes with transfer potential: 72.0%) were more likely to be transferable than genes from Clusters 1–3 (72% of genes in Clusters 4–6 were transferable compared to 13.2% of genes in Clusters 1–3, Figure 4E). Resistance genes in Clusters 1–3 were more frequently observed in *Pseudomonadota* (53.4 ± 20.3%, Figure 4F), as *Pseudomonadota* decreased over time (Figure 1D), it makes sense that these genes would also decrease over time. Genes in Clusters 4–6 were often from *Bacillota* (46.1 ± 17.2%, Figure 4F), *Actinomycetota* (17.3 ± 12.0%), and *Bacteroidota* (17.3 ± 18.3%), so the increase in these genes over time mirrored the increase of the taxa (Figure 1D) that most frequently carried them.

### Infant gut resistome assembly links to shifts in microbial carbohydrate metabolism

**Figure 5:**
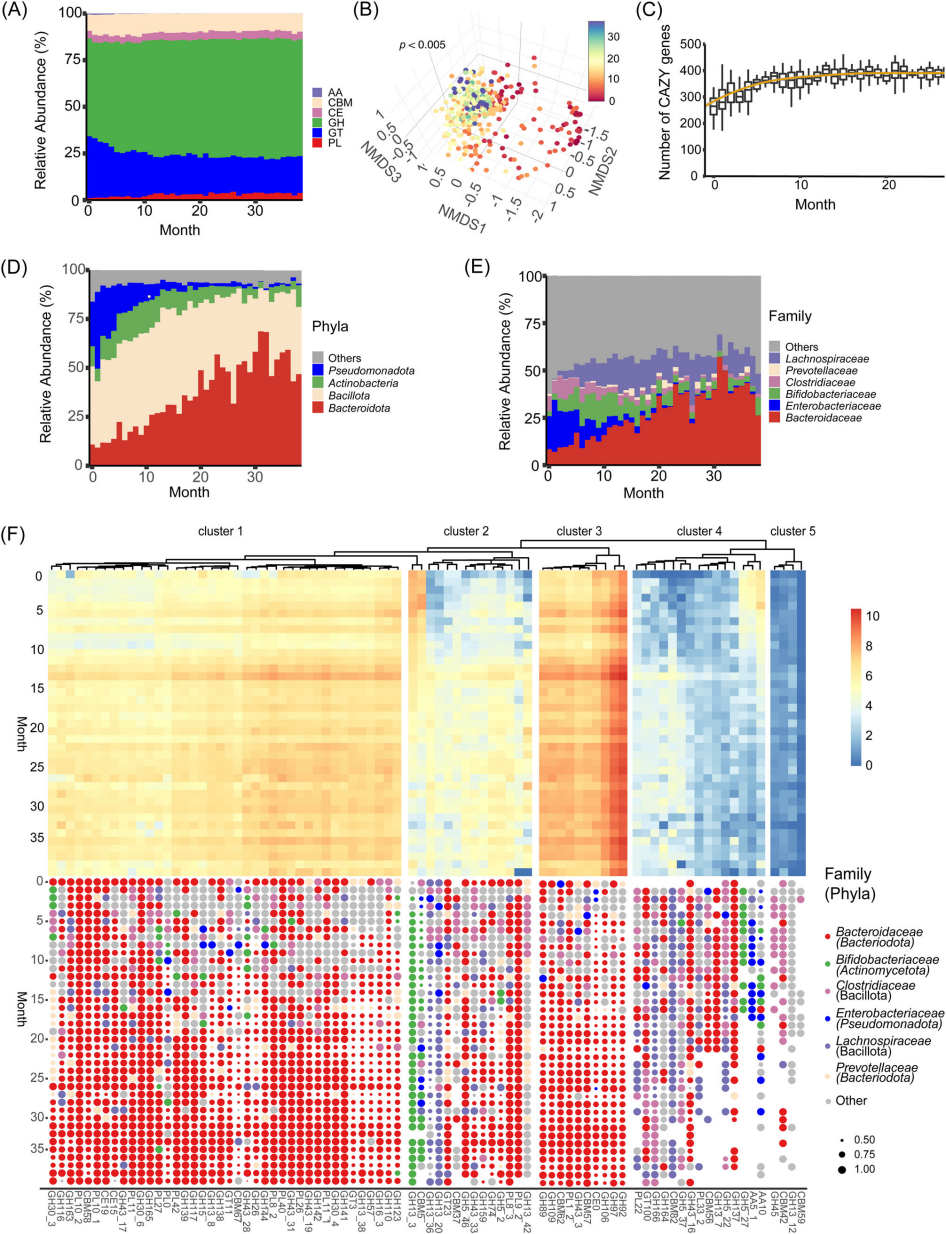
Functional capacity of infants' gut microbiome during early life. (A) Bar plots of the relative abundance of carbohydrate‐active enzymes (CAZy) families per class of enzymes over time. (B) Nonmetric multidimensional scaling (NMDS) of infant gut CAZy results based on a Bray–Curtis dissimilarity calculation (*k* = 4, stress = 0.07). Subsampling to obtain an equal sample size (*N* = 20) at each month (months of sampling size < 20 were deleted) was completed before conducting the ordination analysis and permutational multivariate analysis of variance (PERMANOVA). (C) Box plot demonstrating the richness of observed CAZy enzymes during early infancy. (D, E) Stacked bar plots depicting the predicted microbial hosts of CAZy enzymes at the phyla (D) and family (E) level. (F) Heatmap depicting the abundance of CAZy families, which were significantly associated with infant age. The abundance values were log‐transformed (log [value + 1, 10]) for better visualization. The lower dot plot showed the inferred bacteria hosts producing corresponding CAZy enzymes and the sizes of dots indicated the confidence level (%) of microbial inferences. The absence of a dot indicated that our inability to predict the bacterial origin or the CAZy family was not detected at a particular time. AA, auxiliary activities; CBM, carbohydrate‐binding modules; CE, carbohydrate esterases; GH, glycoside hydrolases; GT, glycosyltransferases; PL, polysaccharide lyases.

To gain insight into the influences of carbohydrate metabolism on resistome, we proceeded with in‐depth functional profiling analyses that observed six classes of carbohydrate‐active enzymes (CAZy) (Table S3). Over 80% of profiled CAZy genes belonged to the glycoside hydrolase or glycosyltransferase classes. Although the relative contribution of glycosyltransferase steadily decreased over time, the levels of glycoside hydrolases increased (Figure 5A). The carbohydrate metabolism genes showed distinct differences over time (PERMANOVA by adonis2 and distance‐based redundancy analysis [db‐RDA], *p* < 0.05, Figure 5B), with the number of distinct CAZy enzymes increasing over time (Figure 5C).

Approximately 22.8% of all CAZy enzymes were present in *Pseudomonadota* at birth, decreasing to only 0.9% of all CAZy enzymes by the age of 3 years. In contrast, 10.9% of CAZy enzymes in the infant gut microbiome at birth were found in *Bacteroidota*, rising to 47.0% at age 3 years (Figure 5D,E). Looking at the samples collected during the first 3 years of life, there were 83 CAZy enzymes in five clusters significantly associated with age, with glycoside hydrolases being the most frequently observed enzyme class (60.3% of enzymes), followed by polysaccharide lyases (PLs) (14.9%), carbohydrate‐binding modules (12.4%), glycosyltransferases (7.4%), carbohydrate esterases (2.5%), and auxiliary activities (2.5%). Overall, enzymes belonging to Clusters 1 and 2 were predominantly found in glycoside hydrolase (GH) and PL families, and their hosts were more diversified at birth, gradually converging towards origins in *Bacteroidaceae* (*Bacteroidota*) with increasing age. The activities observed for the (sub)families from the GH (e.g., β‐1,4‐glucanase and α‐1,4‐glucan) were related to complex carbohydrates present in breast milk and plant‐based foods (Table S3).

Most CAZy enzymes within Cluster 4 were prevalent during the first days of life but decreased at later time points and likely originated from *Enterobacteriaceae* (*Pseudomonadota*). A subset of Cluster 4 enzymes (e.g., CBM82) increased over time; most often, the enzymes that increased over time were predicted to originate in *Lachnospiraceae* and *Clostridiaceae* (*Bacillota*) (Figure 5F). Members of CBM82 were reported to be specifically solid‐food‐related enzymes that possess the β‐1,3‐glucan binding function to plant‐based substrates (e.g., starch) [17] (Table S3).

Furthermore, we examined the association between the 110 age‐associated MEGIDs (ARG IDs in MEGARes, https://www.meglab.org/) and the 83 age‐associated CAZy enzymes and found that half of the possible MEGID‐CAZy pairs (over 5000) were significantly correlated. We then used a multivariate linear mixed‐effect model to test for the association between CAZy enzymes (predictor) and MEGIDs (outcome). We observed that the variation in resistance abundance due to carbohydrate metabolism (40.7%) was greater than the variation due to microbial composition (36.1%). This close connection between resistance was validated by our genome‐centric analysis by functional profiling of metagenome‐assembled genomes (MAGs) from the top 4 genera with the highest abundance of resistance genes (*Escherichia*, *Bifidobacterium*, *Bacteroides*, and *Klebsiella*) with 110 age‐associated MEGIDs and 83 age‐associated CAZy enzymes (Figure S6). The frequency of both resistance genes and CAZy enzymes did vary based on the genus or origin, with resistance genes more frequently observed in *Escherichia* and *Klebsiella* (more prevalent taxa during the first days of life), and CAZy enzymes were more common in *Bacteroides* and *Bifidobacterium* (more prevalent taxa in older infants) (Figure S6). This creates a microbial taxa connection between the gradually modified infant gut microbial carbohydrate metabolism and the reduced prevalence of gut resistance genes, mirroring the transition from low‐CAZy enzyme/high‐resistance genes taxa to high‐CAZy enzyme/low‐resistance genes taxa over time.

### Increasing lateral gene transfer (LGT) events and transferrable resistance genes in infants' gut

**Figure 6:**
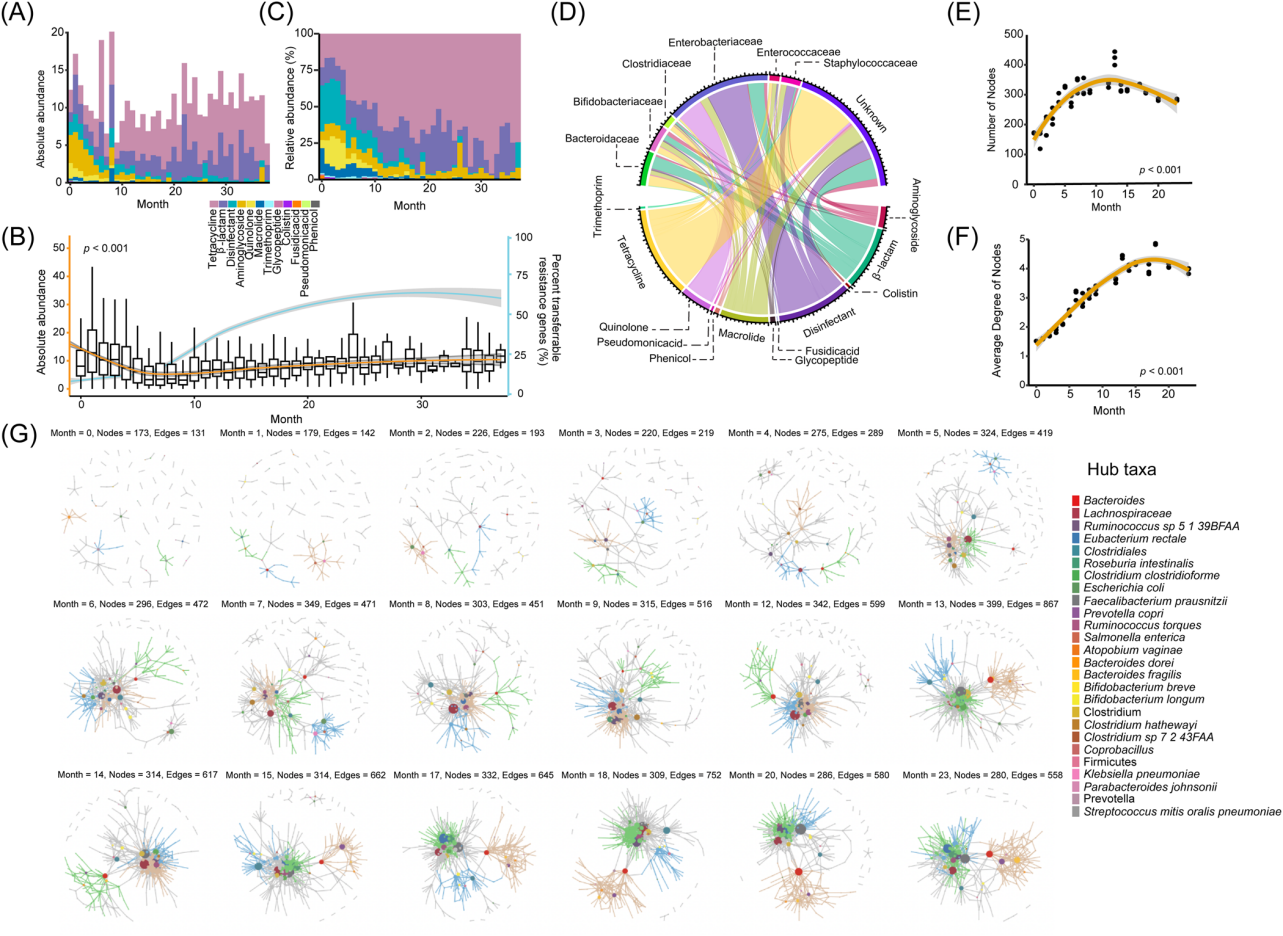
The development of infant gut mobilome. (A) Stacked bar plot depicting the absolute abundance of acquired resistance genes as measured by the copy numbers of resistance RPKG (resistance‐related reads per kilobase per genome equivalent). (B) Box plot demonstrating the dynamic change of the overall resistance. Outliers were eliminated for better visualization. (C) Stacked bar plot depicting the relative abundance of acquired resistance genes as measured at the antimicrobial class level. The local regression line (blue) represents the percentage of transferable genes (ResFinder 4.0) in total resistance genes (MEGARes 2.0). (D) Chord diagram connecting acquired resistance genes and inferred microbial hosts at the family level. Bacterial families and corresponding genes which contributed to <2% of overall resistance were filtered out for better visualization. (E, F) Scatter plots obtained by three independent subsamplings indicate the number of nodes (E) and corresponding average degrees (F) from predicted lateral gene transfer (LGT) networks in the infant's gut over time. (G) Networks illustrating predicted LGT events during early infancy. Subsampling was performed to minimize sample size bias, and time points without sufficient samples (*N* < 40) were removed for this analysis. At each time point, three representative microbial clusters and the corresponding nodes (hub taxa) were colored to depict a gradually modified network; hub taxa were labeled, from top to bottom, with decreased occurrence.

We next examined the prevalence of acquired resistance genes in all infants with ResFinder 4.0. The overall abundance of transferrable genes was significantly associated with infant age (LMM, *p* < 0.001). Starting at ~8.1 RPKG (median; IQR, 9.1) at birth, levels of transferrable genes increased to 12.8 RPKG at Month 1 (IQR, 15.0), then dropped to 3.1 RPKG at Month 9 (IQR, 3.6), and finally stabilizing around 8.2 RPKG (Months 10 to 38) (Figure 6A,B). Consistent with analyses based on the MEGARes database, transferrable tetracycline resistance genes (58.7%) were the most frequently observed resistance genes in the developing infant gut microbiome, followed by β‐lactamases (19.8%), disinfectant resistance genes (8.2%), aminoglycoside resistance genes (6.6%), and quinolone resistance genes (3.4%) (Figure 6C). Consequently, we observed a gradually increased level of acquired resistance genes in infant gut resistomes over time (Figure 6B). *Enterobacteriaceae* was found to be the dominant family carrying acquired resistance genes, followed by *Bacteroidaceae*, *Bifidobacteriacae*, *Staphylococcaceae*, *Clostridiaceae*, and *Enterococcaceae* (Figure 6D).

We further expanded our analysis to predict the occurrence of LGT in infant gut microbiome. Microbes from 172 microbial taxa were involved in either receiving or transmitting ARGs by LGT in newborns, with the number of involved taxa increasing to a peak of 422 taxa at Month 13 and then slightly decreasing at older ages (Figure 6E). As measured by LGT events per microbial taxon, transfer frequency gradually increased over time (Figures 4E and 6F, and Table S4). LGT events were predicted to occur across branches in the phylogenetic tree, 63.5% of transfers occurred across microbial families, with a subset of 4.8% of these transfers likely occurring across microbial phyla (e.g., mobilization among *Actinomycetota*, *Bacillota*, and *Pseudomonadota*) (Figure 6G). Our network modeling identified 26 “hub” taxa that were predicted to have the greatest occurrence of LGT across time points, these taxa included microbes belonging to *Clostridiaceae*, *Enterobacteriaceae*, *Bacteroidaceae*, and *Lachnospiraceae*, *Bifidobacteriaceae*, *Prevotellaceae*, and *Ruminococcaceae* (Figure 6G). All proposed hub taxa have been proven to be frequently associated with LGT events by experimental evidence [18, 19]. Specifically, *Escherichia coli* and *Klebsiella pneumoniae* are well‐known for carrying acquired resistance genes and virulence and can cause severe blood infections that are difficult to treat in infants [20, 21]. Of note, *Bifidobacterium* frequently transferred resistance genes to microbiomes belonging to *Actinobacteria*, *Bacillus*, and *Clostridia* [22]. Consequent to the high levels of LGT, we observed an increasing prevalence of acquired resistance genes in developing infant gut resistome with age as the genes spread across different microbial taxa.

## DISCUSSION

In early life, infants acquire a large resistome burden, and our understanding of the resistome in healthy infants was comparably less known than the gut microbiome. Considering what is known about the microbiome, the gut resistome is relatively better studied in hospitalized infants than in community‐dwelling children [23, 24, 25, 26, 27, 28, 29]; however, healthy infants are also an important reservoir of resistance genes [10, 30, 31]. By defining healthy infants as term, vaginally delivered, and without NEC or any recorded antibiotic use, we systematically examined the natural assembly of the healthy infant gut resistome. Leveraging public metagenomes (*N* = 858 healthy metagenomes), we found that the abundance and richness of resistance genes were the highest for infants at birth and then gradually decreased over time, the progression of which could be distinguished into two phases: the multicompound resistance phase (Months 0–7), and the tetracycline–mupirocin–β‐lactam‐dominant phase (Months 8–14). Even in healthy infants, some genes are resistance genes to clinically important antibiotics (e.g., penams, macrolides, and aminoglycosides) widely distributed in the gut, posing a threat to effective antibiotic treatment. Our findings in healthy infants from industrialized countries defined the natural succession of the gut resistome, providing an essential reference for future potential interventions to reduce ARG carriage in this population.

Our systematic microbial origin analysis identified that microbes belonging to *Escherichia*, *Bifidobacterium*, *Bacteroides*, and *Klebsiella* were the main bacterial hosts of resistance genes, which was consistent with studies indicating that *E. coli* as the main microbe differentiating resistome structure [10, 12, 23, 27, 32, 33]. This is also congruent with an earlier study that found an increased resistance burden in the bifidobacterial community with age [34].

Age exerts a strong influence on the infant's gut resistome. This is consistent with the age‐dependent assembly of the infant microbiota [6, 10, 15], as the microbiome shapes the resistome [12, 32]. Previous studies tried to examine the influence of age on the infant gut resistome but were limited to fewer time points [12, 27, 32], thus limiting the extent of possible longitudinal analysis. In the present assembled cohort, we observed an overall decrease in resistance genes in infants during the first 3 years of life. Despite the reduction trend, the risks from resistance genes in infants remain because the decrease occurs in intrinsic resistance genes while transferable resistance genes generally increase over time. Clinical factors such as study cohort, method of feeding, and NEC were significantly associated with the overall resistance in addition to age, suggesting these factors had an enduring influence on infant gut resistome from 0 to 3 years. For example, Infants with NEC had a higher total abundance of resistance genes.

The assembly of gut microbial communities is often driven by diet (i.e., transition from milk to solid food) during infancy [35, 36, 37]. The infants begin eating solid food at about 6 months and gradually transition to table foods. The changing infant diet includes distinct types of carbohydrates, which call for varied metabolic functions of both the host and the gut microbiome. For example, dietary fiber from solid food is often digested by *Bacteroides* [17, 37]. The assembly of the gut resistome is likely driven by the changing microbial needs for effective metabolism of carbohydrates in infants. The diet changes during infancy are consistent with an increasing need for CAZy from *Bacteroidota* (a relatively small reservoir of ARGs), and a decreasing role for *Pseudomonadota* (often equipped with ARGs), which match our findings in this study. The structures of human milk oligosaccharides require a distinct set of GH, particularly those in GH families GH13 and GH26 that are commonly found in Bacteroidetes and Bifidobacterium [18], which are aligned with our result.

Furthermore, resistance genes with transfer potential threaten public health more than intrinsic resistance genes [38, 39, 40, 41]. Previous studies have indicated that infants have a 10‐fold greater rate of evolution and strain turnover and higher abundances of ARGs on mobile genetic elements than adults [28, 29, 42]. Our mobilome analysis observed a gradual increase in acquired resistance genes (via ResFinder) in infants’ gut despite the reduction of the overall resistome with age. With the LGT analysis, we observed an increasing transfer frequency among microbial clades from Month 0 to Month 23. The majority of LGT occurred across bacterial families (>60%), with a subset of transfer events occurring across phyla (~5%) (Figure 6), even though the major determinant of mobile resistance was bacterial phylogeny [43]. This was likely a result of the microbial community changing with increased exposure to the environment and a more complex diet as the infants aged, consistent with a recent study that found an elevated rate of gene transfer in the industrialized human microbiome [44].

Although informative, combining metagenome data from different studies without experimental validation consistent across cohorts was a major limitation of this work. The lack of standardized protocols (for sample collection, DNA extraction, sequencing platform), variability in study populations, and absence of more detailed information regarding the sample host (such as maternal nutrition status, medical history, and other potential factors) further complicated the data integration. Future studies employing greater consistency and validation across cohorts are needed to decipher further the relationship between dietary transitions and the infant gut resistome. Despite the limitations of this work, this study still provides insight into the changes that occur in the gut resistome as infants age and suggests a role for diet in shaping the infant resistome.

## CONCLUSIONS

In conclusion, this study provided valuable insights into the natural assembly of the healthy infant gut resistome and the factors that influence the resistome. Our findings revealed that resistance genes are enriched early in life, with the highest abundance and richness observed at birth and gradually decreasing over time. Additionally, the age‐related changes in the infant gut resistome were influenced by the changes in microbial carbohydrate metabolism necessitated by changes in the infant diet and clinical factors. The study also highlighted a gradual increase in acquired resistance genes in the infant gut resistome over time, corresponding with an increase in LGT events.

## METHODS

### Global metagenome collection

To identify publically available datasets useful for our study of the assembly of the infant gut resistome we conducted a series of literature searches using keywords such as ((microbiome OR microbiota) AND (infant OR neonatal OR neonate* OR newborn OR “preterm infant”) AND (feces OR stool OR fecal OR feces OR gut)) in PubMed (2003), Web of Science (3319), and Scopus (1628) between the years 2015 and 2020 (Figure S7). A total of 6909 relevant articles were retrieved, out of which 3475 were identified as nonduplicate articles. We then selected studies with complete metagenomic sequencing profiles and metadata in publicly available data repositories that had sample collection during the first 1120 days of life. This led us to 17 studies with data available for this work. These 17 studies had data on 4132 metagenomes from 963 infants and 19 cohorts available across multiple data depositories (i.e., the Sequence Read Archive, the European Nucleotide Archive, and local servers; Table S1). All relevant metadata was compiled and is available in Table S1. The accessed metagenomes included infants from six countries (i.e., United States, Sweden, Finland, Estonia, Russia, and Italy) and were sequenced on multiple platforms, including several Illumina platforms (i.e., HiSeq. 2000, HiSeq. 2500, NextSeq. 500, MiSeq, and HiSeq. 4000; Table S1). Greater than 13,234 Gb raw shotgun metagenomic sequences were obtained, representing the largest assembled cohort of infant gut metagenomes for a single analysis. We used this data to explore the trajectory of the infant resistome during the first 1000 days, including the interaction of the resistome with related confounding variables. All samples involved in this work had a sequencing depth of over five million cleaned reads to ensure the capture of rare ARGs per a published standard [45].

### Sequence preprocessing and quality control

We applied unified quality controls to all samples from all cohorts before any downstream analyses. Host sequences were identified by aligning metagenomes to the human reference genome GRCh38, by using BMtagger in bmtools (v1), and were removed. The remaining reads were trimmed using Trimmomatic (v0.36) to remove low‐quality sequences. Trimming was done with parameters (LEADING:3 TRAILING:3 SLIDINGWINDOW: 4:15) and was adjusted for sequences ≥150 bp (MINLEN:99) and 100 bp (MINLEN:40). FastUniq (v1.1) was next applied to remove duplicate reads, and the resulting sequences were sorted to match pairs using bbmap (repair.sh, v38.72). Quality control was performed before and after the sequence trimming for direct comparisons to track the sequence's “improvement” during data processing. Briefly, bbmap was used to assess sequencing depth and read length, and fastqc (v0.11.5) was applied to examine the sequence quality with default settings (Figure S1). A direct comparison of sequences during data pre‐processing indicated that the number of reads of infant gut metagenomes was ~7.6 million (median; IQR, 11.4 million) and was decreased to 5.7 million (median; IQR, 10.6 million) after quality control (pair Wilcoxon test, *p* < 0.001 in all cases) (Figure S1).

### Taxonomic profiling and metagenome assembly

Sorted sequences were used for taxonomic profiling via MetaPhlAn2 [46] (v2.7.7) and data were analyzed at the phyla, family, and species levels. MEGAHIT [47] (v1.1.1) was used to assemble metagenomes per sample with default parameters. We modified the reported names of phyla to match the updated official nomenclature [48].

### Resistome analysis

MEGARes [49] 2.0, which contains genes conferring resistance to all types of antimicrobial compounds (i.e., antibiotics, heavy metals, biocides, and multicompounds), was used to profile the infant resistome. Resistance genes in MEGARes 2.0 were classified at five levels, from top to bottom: the “Type” of a compound to which the accession confers resistance (i.e., drug, biocide, metal, and multicompound), the “Class” of antimicrobial compounds to which a gene confers resistance (e.g., aminoglycosides, and biocide, and metal resistance), the “Mechanism” by which this resistance is conferred, the “Group” name of the genes (e.g., *BLAZ*, and *CTX*), and the “MEGID” for each individual gene accession (e.g., MEG7183 and MEG5321). A total of 4238 individual resistance genes were mapped (of 7378 genes from MEGARes 2.0 after removal of speculated resistance genes, which required further SNP validation for point mutations in our analyses), representing 143 unique (of 220 in MEGARes V2) mechanisms of resistance, and conferring resistance to four types of compounds (i.e., antibiotics, biocides, heavy metals, and multicompounds). bwa‐mem (v2) was used to align metagenomes to MEGARes 2.0 with default parameters, and resistome analyzer (https://github.com/cdeanj/resistomeanalyzer) was used to analyze the aligned SAM files. Normalization was performed by calculating the number of resistance genes per estimated genome by using a custom script adapted from Li et al. [50, 51, 52]. Genome equivalents were obtained by analyzing sorted sequences via MicrobeCensus (v2.15) with adjusted parameters (‐n 100000000) to use all available sequences [53].

### Microbial hosts of resistance genes

The microbial origin of resistance genes was estimated by assigning taxonomy to assembled contigs harboring target genes. Following alignment, the SAM file was parsed, and the successfully aligned reads harboring resistance genes were extracted and further used to align back to contigs [54]. The resulting resistance‐containing contigs were assigned a taxon via kaiju [55].

### Functional capacity profiling analysis

To study the functional carbohydrate profile of infant gut microbial communities, we used CAZy database [56] as a reference database via DIAMOND (v2.0.15, cutoff E‐value ≤ 10^−10^) [57]. Sequencing reads that were predicted to encode CAZy enzymes were aligned to metagenomic‐assembled contigs by using BWA‐MEM. Contigs carrying sequences that encoded age‐associated CAZy enzymes were further used to be annotated by assigning taxa via kaiju [55].

### Assessing the association between resistome and metabolome by genomic‐centric analysis

We examined the MEGIDs and CAZy enzymes through genomic‐centric analysis on the top 4 genera (*Escherichia*, *Bifidobacterium*, *Klebsiella*, and *Bacteroides*) (Figure 3C). Specifically, a total of 32,277 MAGs were obtained from the research of Zeng et al. [58] and were filtered to keep the MAGs (*N* = 14,280) retrieved from the same set of samples used in our analysis. Assembled genomes were searched for sequence similarity to annotated ARGs present in the MEGAResv2.0 using BLASTN (version 2.12.0) with a coverage threshold of 80%. CAZy enzyme was also profiled in MAGs by aligning to the CAZy database via Diamond. MAGs were taxonomically classified using the Genome Taxonomy Database Toolkit (GTDB‐Tk, v1.7.0) and the “classify_wf” function with default parameters.

### Statistical analysis

Statistical analyses were performed using R (v3.6.2). α‐diversity was measured by the Shannon index for both bacterial species and resistance genes. β‐diversity of microbial species and resistance genes from healthy infants, and CAZy enzyme results from all the infants were generated via Bray–Curtis dissimilarity, and nonmetric multidimensional scaling was afterward used to visualize the compositional variances across these community data by controlling a stress value < 0.1 under four dimensions [59]. For PERMANOVA, we checked for dispersion differences across months based on Bray‐Curtis dissimilarity measures before completing the PERMANOVA with adonis2 in the *vegan* package to test for differences in β‐diversity across months. To address the bias from the study cohort, we further conducted a db‐RDA using the *vegan* package and got the same result. We applied a subsampling strategy to obtain an equal size of samples across months, and only one sample of infants was used in PERMANOVA and db‐RDA. The trajectory of α‐diversity was estimated via a LMM using the R package *lme4* with “age of infants” and “study cohort” as fixed effects and “infant ID” as a random effect.

### Influences of clinical variables on the gut resistome

LMMs were also used to assess the statistical impact of clinical variables on the infant resistome. Resistance values were standardized via an inverse‐normal transformation, and a variance inflation factor was used to identify collinearity between variables. The final model was as below, and the response $Y_{\mathrm{resistance}}$ represented the observed value (we took the overall resistance value, the sum of four types of resistance, here as an example):

(1) $$\begin{array}{ccl} Y_{\mathrm{resistance}} & = & \beta_{1}\left(\mathrm{Infant}\;\mathrm{age}\right)+\beta_{2}\left(\mathrm{Geography}\right)\\ & & +\beta_{3}\left(\mathrm{Antibiotics}\right)+\beta_{4}\left(\mathrm{Delivery}\;\mathrm{mode}\right)\\ & & +\beta_{5}\left(\mathrm{Feeding}\right)+\beta_{6}\left(\mathrm{Term}\right)+\beta_{7}\left(\mathrm{NEC}\right)\\ & & +\beta_{8}\left(\mathrm{Study}\;\mathrm{cohort}\;\mathrm{ID}\right)\\ & & +\left(1\right|\mathrm{subject\_ID}). \end{array}$$

The resistance values of drug, biocide, metal, and multi‐compound were considered, respectively. The influence of fixed effects on resistome was calculated via marginal *R* ^2^, where $\sigma_{f}^{2}$ is the variance of fixed effects, $\sigma_{\alpha }^{2}$ is between‐subject variance, and $\sigma_{\varepsilon }^{2}$ is the residual variance. To further quantify the contribution of each predictor to the variance of resistome, variable explainability was calculated as below [60]:

$$R_{\mathrm{mi}}^{2}=\frac{\sigma_{f}^{2}}{\sigma_{f}^{2}+\sigma_{\alpha }^{2}+\sigma_{\varepsilon }^{2}}\;*\;\frac{f\_variance\_i}{\sum_{i=1}^{N}f\_variance\_i},$$

where *f_variance_i* is the variance of the *i*th fixed variable, and r variance explained is the variance explained by random effect.

### Age‐associated MEGIDs and CAZy enzymes

We also used GLMM to identify age‐associated MEGIDs and CAZy enzymes. The fixed effect and random effect were the same as the Equation (1) and the dependent variable was *Y*(_MEGID_) or *Y*(_CAZy enzyme_), followed by multiple comparison corrections. Considering the count data of MEGID contained excess zero, we further categorized each gene into binary outcomes (0 or 1). Significant age‐associated MEGIDs and CAZy enzymes were chosen by age (*p*_adj_ < 0.001 with Bonferroni correction) and a total of 110 MEGIDs and 83 CAZy enzymes were obtained and grouped into clusters by calculating the Euclidean distance. We further compared all the pairs of the associations between 83 age‐associated CAZy enzymes and 110 age‐associated MEGIDs using LMM. We employed each CAZy enzyme, alongside identical fixed and random effects in the Equation (1), as predictor variables, with each MEGID serving as the response variable. We also compared all the pairs of the associations between 111 resistomes from the mechanism level and 6 CAZy groups (AA, GH, GT, PL, CE, and CBM) from a general perspective.

### Microbiota and CAZy function ability to explain the infant gut resistome

To model the effect of clinical variables and microbiota at genus level or CAZy enzyme function on the resistome, multivariate LMMs of the following form were used [61]:

(2) $$\begin{array}{ccl} y_{aijkmlqps} & = & {\text{Infant age}}_{a}+{\text{Geography}}_{j}\\ & & +{\text{Delivery Mode}}_{k}+{\text{Term}}_{m}\\ & & +{\text{Feeding}}_{l}+{\text{Antibiotics}}_{q}+{\text{NEC}}_{p}\\ & & +{\text{Study cohort}}_{s}+m_{i}{+e}_{ajkmlqpsi}. \end{array}$$

In Equation (2), $y_{\mathrm{aijkmlqps}}$ is the summed resistance value. $m_{i}$ is the random effect accounting for multiple samples from the *i*th infant ~ NID (0, *σ* ^2^_*g*_M), where $\sigma_{g}^{2}$ is the microbial variance and *M* is the genus relation matrix [62, 63]. The genus relation matrix *M* was calculated as:

$$M=\frac{XX^{\prime }}{n},$$

where *X* is the matrix of normalized bacterial relative abundance and *n* is the number of microbes. The $e_{\mathrm{ajkmlqpsi}}$ is residual variable. We used a second LMM to estimate the CAZy enzyme influence on variation in the resistance. The model was similar to (2), but M was replaced with C, and the random effect $c_{i}$ ~ NID (0, *σ* ^2^_c_C). The CAZy enzyme relation matrix C was similar to M which was computed as a variance–covariance matrix from CAZy enzyme composition. The resistance variances explained by the microbiome and CAZy enzyme were estimated by

$$\frac{\sigma_{g}^{2}}{\sigma_{p}^{2}},\frac{\sigma_{c}^{2}}{\sigma_{p}^{2}},$$

where $\sigma_{p}^{2}$ is the overall resistance variance.

### Prediction of infant age by resistome using RFs

RFs were used to predict infant age from the absolute abundance of MEGIDs since this approach can detect both linear and nonlinear relationships between resistance and infant age in days. We split the data based on infants in a 7:3 ratio (training set:test set) by each cohort to prevent bias from different proportions of each study cohort present in the training and testing set. The number of predictors to be used in the RF model was determined through 10‐fold cross‐validation over 100 iterations. The number of trees, variables for each split (mtry), and the total number of MEGIDs (node size) to grow the forest were set to 500, 35, and 35, respectively. Default selections were retained for the remaining hyperparameters. In the main model, we considered not only MEGID but also infant host ID to account for repeated measures, and study cohort to address any remaining bias from the differences in each cohort. Of note, we had over 900 infants with samples included in this study and performed a 1:*n* numeric assignment for infant ID randomly. In an additional sensitivity analysis, we only considered the impact of MEGID (the result is shown in Table S2).

We used the percentage increase in mean squared error (Inc% mean squared error) with the out‐of‐bag samples to rank the importance of each MEGID in predicting infant age. Further, we used feature contributions estimated by *forestfloor*, which shows how the predicted infant age changes with each predictor. Out‐of‐bag observations were used to minimize overfitting when estimating the marginal effects.

### DMM clustering of infant gut microbiome and resistome

DMM model is integrated to predict the structure of healthy infant gut microbiomes and resistomes using the R package *DirichletMultinomial* as described in previous studies [6, 64]. The best‐fit number of DMM components was based on the minimum Laplace approximation score of 1 to 15. DMM modeling reduces the effect of a small sample size on a rich community where the clusters will be biased towards extreme sample sizes.

### Transferrable resistance genes and LGT in infant gut microbiomes

We specifically analyzed the acquired infant gut resistome by aligning metagenomic sequences to ResFinder (v4.1) using bwa‐mem (v2) [65]. Bedtools was used to analyze the alignments with a coverage cut‐off of 80%. Resultant sequences were normalized in the same pipeline with MEGARes 2.0 analysis. The LGT events in infant gut microbiomes were predicted via WAAFLE (http://huttenhower.sph.harvard.edu/waafle) and the frequency of LGT was normalized to per 1 K assembled genes before any downstream comparisons. The LGT networks were constructed using the predicted edge matrixes and were visualized using the R package igraph (v1.2.11).

## AUTHOR CONTRIBUTIONS

Xinming Xu, Qingying Feng, and Jinxin Liu conceived and designed the analysis, collected the data, and wrote the paper. Xinming Xu, Qingying Feng, and Yunlong Gao performed the analysis. Qu Cheng, Ke Xu, Yucan Li, and Kelin Xu contributed to the methodology. Wanqiu Zhang, Nhu Nguyen, Diana H. Taft, David A. Mills, Danielle G. Lemay, Weiyun Zhu, Shengyong Mao, and Anyun Zhang reviewed the manuscript. Xinming Xu, Qingying Feng, Tao Zhang, and Jinxin Liu revised the paper. David A. Mills and Jinxin Liu provided the resources. Kelin Xu and Jinxin Liu supervised the project. All authors have read the final manuscript and approved it for publication.

## CONFLICT OF INTEREST STATEMENT

David A. Mills is a cofounder of Evolve Biosystems and BCD Biosciences. Evolve Biosystems and BCD Biosciences had no role in this manuscript's conceptualization, design, data collection, analysis, or preparation. The remaining authors declare no conflict of interest.

## ETHICS STATEMENT

No animals or humans were involved in this study.

## Supporting information


**Figure S1**: The attributions (counts, lengths, and qualities) of raw and processed sequencing reads.
**Figure S2**: Sankey diagram connecting resistance genes from month 0‐14 at the antimicrobial compound class level (left) to the predicted bacterial hosts at the genus (middle) and phylum level (right).
**Figure S3**: The dynamics of resistome in *Bifidobacterium* and *Escherichia* genome.
**Figure S4**: Clinical variables significantly associated with the inverse‐normal transformed absolute abundance of summed resistance.
**Figure S5**: Forest floor main effect plots of random forest mapping structure of model predicting panel ratings of infant age on basis of MEGID.
**Figure S6**: Heatmap of age‐associated MEGID and CAZy enzymes mapped in certain genus of metagenome‐assembled genomes (MAGs).
**Figure S7**: Flow chart of systematic review and selection process.

**Table S1**: Descriptive characteristics of metagenomes involved in this study.
**Table S2**: Detailed information of ranked lists of the top MEGIDs (resistance).
**Table S3**: Function and predicted microbial host of 83 age‐dependent CAZy.
**Table S4**: Transfer potential information regarding age‐dependent MEGIDs.

## Data Availability

The raw sequencing reads for the metagenomic samples used in this study were downloaded from previously published projects, including: Bäckhed et al. (2015) (BioProject #PRJEB6456), Raveh‐Sadka et al. (2015) (BioProject #PRJNA273761), Raveh‐Sadka et al. (2016) (BioProject #PRJNA294605), Gibson et al. (2016) (BioProject #PRJNA301903), Ward et al. (2016) (BioProject #PRJNA63661), Vatanen et al. (2016) (BioProject #PRJNA290380), Yassour et al. (2016) (BioProject #PRJNA290381), Asnicar et al. (2017) (BioProject #PRJNA339914), Brooks et al. (2017) (BioProject #PRJNA376580 and PRJNA376566), Chu et al. (2017) (BioProject #PRJNA322188), Olm et al. (2017) (BioProject #PRJNA327106), Pärnänen et al. (2018) (BioProject #PRJNA384716), Ferretti et al. (2018) (BioProject #PRJNA352475), Baumann‐Dudenhoeffer et al. (2018) (BioProject #PRJNA473126), Casaburi et al. (2019) (BioProject #PRJNA390646), Gasparrini et al. (2019) (BioProject #PRJNA489090), and Yassour et al. (2019) (BioProject #PRJNA475246). The python custom scripts used in the upstream metagenomics analysis workflow, including the normalization of resistome, and bacterial origin of resistance, were freely available at https://github.com/cookiemonsterxxm/infantresistome. Supporting Information (figures, tables, scripts, graphical abstract, slides, videos, Chinese translated version, and update materials) may be found in the online DOI or iMeta Science http://www.imeta.science/.

## References

[1] Round, June L. , and Sarkis K. Mazmanian . 2009. “The Gut Microbiota Shapes Intestinal Immune Responses During Health and Disease.” Nature Reviews Immunology 9: 313–323. 10.1038/nri2515 PMC4095778 19343057

[2] Depner, Martin , Diana H. Taft , Pirkka V. Kirjavainen , Karen M. Kalanetra , Anne M. Karvonen , Stefanie Peschel , Elisabeth Schmausser‐Hechfellner , et al. 2020. “Maturation of the Gut Microbiome During the First Year of Life Contributes to the Protective Farm Effect on Childhood Asthma.” Nature Medicine 26: 1766–1775. 10.1038/s41591-020-1095-x 33139948

[3] de Muinck, Eric J. , and Pål Trosvik . 2018. “Individuality and Convergence of the Infant Gut Microbiota During the First Year of Life.” Nature Communications 9: 2233. 10.1038/s41467-018-04641-7 PMC5993781 29884786

[4] Tamburini, Sabrina , Nan Shen , Han C. Wu , and Jose C. Clemente . 2016. “The Microbiome in Early Life: Implications for Health Outcomes.” Nature Medicine 22: 713–722. 10.1038/nm.4142 27387886

[5] Walker, W. Allan . 2017. “The Importance of Appropriate Initial Bacterial Colonization of the Intestine in Newborn, Child, and Adult Health.” Pediatric Research 82: 387–395. 10.1038/pr.2017.111 28426649 PMC5570628

[6] Stewart, Christopher J. , Nadim J. Ajami , Jacqueline L. O'Brien , Diane S. Hutchinson , Daniel P. Smith , Matthew C. Wong , Matthew C. Ross , et al. 2018. “Temporal Development of the Gut Microbiome in Early Childhood From the TEDDY Study.” Nature 562: 583–588. 10.1038/s41586-018-0617-x 30356187 PMC6415775

[7] Rothschild, Daphna , Omer Weissbrod , Elad Barkan , Alexander Kurilshikov , Tal Korem , David Zeevi , Paul I. Costea , et al. 2018. “Environment Dominates Over Host Genetics in Shaping Human Gut Microbiota.” Nature 555: 210–215. 10.1038/nature25973 29489753

[8] Rutayisire, Erigene , Kun Huang , Yehao Liu , and Fangbiao Tao . 2016. “The Mode of Delivery Affects the Diversity and Colonization Pattern of the Gut Microbiota During the First Year of Infants' Life: A Systematic Review.” BMC Gastroenterology 16: 86. 10.1186/s12876-016-0498-0 27475754 PMC4967522

[9] Vatanen, Tommi , Aleksandar D. Kostic , Eva d'Hennezel , Heli Siljander , Eric A. Franzosa , Moran Yassour , Raivo Kolde , et al. 2016. “Variation in Microbiome LPS Immunogenicity Contributes to Autoimmunity in Humans.” Cell 165: 842–853. 10.1016/j.cell.2016.04.007 27133167 PMC4950857

[10] Backhed, Fredrik , Josefine Roswall , Yangqing Peng , Qiang Feng , Huijue Jia , Petia Kovatcheva‐Datchary , Yin Li , et al. 2015. “Dynamics and Stabilization of the Human Gut Microbiome During the First Year of Life.” Cell Host & Microbe 17: 690–703. 10.1016/j.chom.2015.04.004 25974306

[11] Stiemsma, Leah T. , and Stuart E. Turvey . 2017. “Asthma and the Microbiome: Defining the Critical Window in Early Life.” Allergy, Asthma & Clinical Immunology 13: 3. 10.1186/s13223-016-0173-6 PMC5217603 28077947

[12] Li, Xuanji , Jakob Stokholm , Asker Brejnrod , Gisle A. Vestergaard , Jakob Russel , Urvish Trivedi , Jonathan Thorsen , et al. 2021. “The Infant Gut Resistome Associates With *E. coli*, Environmental Exposures, Gut Microbiome Maturity, and Asthma‐Associated Bacterial Composition.” Cell Host & Microbe 29: 975–987.e974. 10.1016/j.chom.2021.03.017 33887206

[13] Laxminarayan, Ramanan , Precious Matsoso , Suraj Pant , Charles Brower , John‐Arne Røttingen , Keith Klugman , and Sally Davies . 2016. “Access to Effective Antimicrobials: A Worldwide Challenge.” The Lancet 387: 168–175. 10.1016/S0140-6736(15)00474-2 26603918

[14] Xiao, Liwen , Jinfeng Wang , Jiayong Zheng , Xiaoqing Li , and Fangqing Zhao . 2021. “Deterministic Transition of Enterotypes Shapes the Infant Gut Microbiome at an Early Age.” Genome Biology 22: 243. 10.1186/s13059-021-02463-3 34429130 PMC8383385

[15] Subramanian, Sathish , Sayeeda Huq , Tanya Yatsunenko , Rashidul Haque , Mustafa Mahfuz , Mohammed A. Alam , Amber Benezra , et al. 2014. “Persistent Gut Microbiota Immaturity in Malnourished Bangladeshi Children.” Nature 510: 417–421. 10.1038/nature13421 24896187 PMC4189846

[16] Vaz, Louise E. , Kenneth P. Kleinman , Marsha A. Raebel , James D. Nordin , Matthew D. Lakoma , M. Maya Dutta‐Linn , and Jonathan A. Finkelstein . 2014. “Recent Trends in Outpatient Antibiotic Use in Children.” Pediatrics 133: 375–385. 10.1542/peds.2013-2903 24488744 PMC3934343

[17] Ye, Lingqun , Promi Das , Peishun Li , Boyang Ji , and Jens Nielsen . 2019. “Carbohydrate Active Enzymes are Affected by Diet Transition From Milk to Solid Food in Infant Gut Microbiota.” FEMS Microbiology Ecology 95: fiz159. 10.1093/femsec/fiz159 31589310

[18] Shoemaker, N. B. , H. Vlamakis , K. Hayes , and A. A. Salyers . 2001. “Evidence for Extensive Resistance Gene Transfer Among Bacteroides spp. and Among Bacteroides and Other Genera in the Human Colon.” Applied and Environmental Microbiology 67: 561–568. 10.1128/AEM.67.2.561-568.2001 11157217 PMC92621

[19] Price, Morgan N. , Paramvir S. Dehal , and Adam P. Arkin . 2008. “Horizontal Gene Transfer and the Evolution of Transcriptional Regulation in *Escherichia coli* .” Genome Biology 9: R4. 10.1186/gb-2008-9-1-r4 18179685 PMC2395238

[20] Wyres, Kelly L. , Ryan R. Wick , Louise M. Judd , Roni Froumine , Alex Tokolyi , Claire L. Gorrie , Margaret M. C. Lam , et al. 2019. “Distinct Evolutionary Dynamics of Horizontal Gene Transfer in Drug Resistant and Virulent Clones of *Klebsiella pneumoniae* .” PLoS Genetics 15: e1008114. 10.1371/journal.pgen.1008114 30986243 PMC6483277

[21] Lou, Yue C. , Matthew R. Olm , Spencer Diamond , Alexander Crits‐Christoph , Brian A. Firek , Robyn Baker , Michael J. Morowitz , and Jillian F. Banfield . 2021. “Infant Gut Strain Persistence is Associated With Maternal Origin, Phylogeny, and Traits Including Surface Adhesion and Iron Acquisition.” Cell Reports Medicine 2: 100393. 10.1016/j.xcrm.2021.100393 34622230 PMC8484513

[22] Deb, Sushanta . 2022. “Pan‐Genome Evolution and its Association With Divergence of Metabolic Functions in *Bifidobacterium* Genus.” World J Microbiol Biotechnol 38: 231. 10.1007/s11274-022-03430-1 36205822

[23] Gibson, Molly K. , Bin Wang , Sara Ahmadi , Carey‐Ann D. Burnham , Phillip I. Tarr , Barbara B. Warner , and Gautam Dantas . 2016. “Developmental Dynamics of the Preterm Infant Gut Microbiota and Antibiotic Resistome.” Nature Microbiology 1: 16024. 10.1038/nmicrobiol.2016.24 PMC5031140 27572443

[24] Raveh‐Sadka, Tali , Brian Firek , Itai Sharon , Robyn Baker , Christopher T. Brown , Brian C. Thomas , Michael J. Morowitz , and Jillian F. Banfield . 2016. “Evidence for Persistent and Shared Bacterial Strains Against a Background of Largely Unique Gut Colonization in Hospitalized Premature Infants.” The ISME Journal 10: 2817–2830. 10.1038/ismej.2016.83 27258951 PMC5148203

[25] Raveh‐Sadka, Tali , Brian C. Thomas , Andrea Singh , Brian Firek , Brandon Brooks , Cindy J. Castelle , and Itai Sharon , et al. 2015. “Gut Bacteria are Rarely Shared by Co‐Hospitalized Premature Infants, Regardless of Necrotizing Enterocolitis Development.” Elife 4: e05477. 10.7554/eLife.05477 25735037 PMC4384745

[26] Ward, Doyle V. , Matthias Scholz , Moreno Zolfo , Diana H. Taft , Kurt R. Schibler , Adrian Tett , Nicola Segata , and Ardythe L. Morrow . 2016. “Metagenomic Sequencing With Strain‐Level Resolution Implicates Uropathogenic *E. coli* in Necrotizing Enterocolitis and Mortality in Preterm Infants.” Cell Reports 14: 2912–2924. 10.1016/j.celrep.2016.03.015 26997279 PMC4819403

[27] Gasparrini, Andrew J. , Bin Wang , Xiaoqing Sun , Elizabeth A. Kennedy , Ariel Hernandez‐Leyva , I. Malick Ndao , Phillip I. Tarr , Barbara B. Warner , and Gautam Dantas . 2019. “Persistent Metagenomic Signatures of Early‐Life Hospitalization and Antibiotic Treatment in the Infant Gut Microbiota and Resistome.” Nature Microbiology 4: 2285–2297. 10.1038/s41564-019-0550-2 PMC6879825 31501537

[28] Casaburi, Giorgio , Rebbeca M. Duar , Daniel P. Vance , Ryan Mitchell , Lindsey Contreras , Steven A. Frese , Jennifer T. Smilowitz , and Mark A. Underwood . 2019. “Early‐Life Gut Microbiome Modulation Reduces the Abundance of Antibiotic‐Resistant Bacteria.” Antimicrobial Resistance & Infection Control 8: 131. 10.1186/s13756-019-0583-6 31423298 PMC6693174

[29] Pärnänen, Katariina , Antti Karkman , Jenni Hultman , Christina Lyra , Johan Bengtsson‐Palme , D. G. Joakim Larsson , Samuli Rautava , et al. 2018. “Maternal Gut and Breast Milk Microbiota Affect Infant Gut Antibiotic Resistome and Mobile Genetic Elements.” Nature Communications 9: 3891. 10.1038/s41467-018-06393-w PMC6155145 30250208

[30] Asnicar, Francesco , Serena Manara , Moreno Zolfo , Duy Tin Truong , Matthias Scholz , Federica Armanini , and Pamela Ferretti , et al. 2017. “Studying Vertical Microbiome Transmission From Mothers to Infants by Strain‐Level Metagenomic Profiling.” mSystems 2: e00164‐16. 10.1128/mSystems.00164-16 28144631 PMC5264247

[31] Ferretti, Pamela , Edoardo Pasolli , Adrian Tett , Francesco Asnicar , Valentina Gorfer , Sabina Fedi , Federica Armanini , et al. 2018. “Mother‐to‐Infant Microbial Transmission From Different Body Sites Shapes the Developing Infant Gut Microbiome.” Cell Host & Microbe 24: 133–145 e135. 10.1016/j.chom.2018.06.005 30001516 PMC6716579

[32] Lebeaux, Rebecca M. , Modupe O. Coker , Erika F. Dade , Thomas J. Palys , Hilary G. Morrison , Benjamin D. Ross , Emily R. Baker , et al. 2021. “The Infant Gut Resistome is Associated With *E. coli* and Early‐Life Exposures.” BMC Microbiology 21: 201. 10.1186/s12866-021-02129-x 34215179 PMC8252198

[33] Yassour, Moran , Tommi Vatanen , Heli Siljander , Anu‐Maaria Hämäläinen , Taina Härkönen , Samppa J. Ryhänen , Eric A. Franzosa , et al. 2016. “Natural History of the Infant Gut Microbiome and Impact of Antibiotic Treatment on Bacterial Strain Diversity and Stability.” Science Translational Medicine 8: 343ra381. 10.1126/scitranslmed.aad0917 PMC5032909 27306663

[34] Duranti, Sabrina , Gabriele A. Lugli , Leonardo Mancabelli , Francesca Turroni , Christian Milani , Marta Mangifesta , and Chiara Ferrario , et al. 2017. “Prevalence of Antibiotic Resistance Genes Among Human Gut‐Derived Bifidobacteria.” Applied and Environmental Microbiology 83: e02894‐16. 10.1128/AEM.02894-16 27864179 PMC5244288

[35] Randa, Saadeh , and Martinez José . 2000. “Complementary Feeding: Family foods for breastfed children” in Development, pp. 1–56. World Health Organization.

[36] World Health Organization . *Complementary feeding: Global*. 2020. Available from: https://www.who.int/health-topics/complementary-feeding#tab=tab_2

[37] EFSA Panel on Nutrition, Novel Foods, Food Allergens , Jacqueline Castenmillerde , Stefaan de Henauw , Karen‐Ildico Hirsch‐Ernst , John Kearney , Helle K. Knutsen , Alexandre Maciuk , Inge Mangelsdorf , Harry J. McArdle , et al. 2019. “Appropriate Age Range for Introduction of Complementary Feeding Into an Infant's Diet.” EFSA Journal 17: e05780. 10.2903/j.efsa.2019.5780 32626427 PMC7009265

[38] Crofts, Terence S. , Andrew J. Gasparrini , and Gautam Dantas . 2017. “Next‐Generation Approaches to Understand and Combat the Antibiotic Resistome.” Nature Reviews Microbiology 15: 422–434. 10.1038/nrmicro.2017.28 28392565 PMC5681478

[39] Partridge, Sally R. , Stephen M. Kwong , Neville Firth , and Slade O. Jensen . 2018. “Mobile Genetic Elements Associated With Antimicrobial Resistance.” Clinical Microbiology Reviews 31: e00088‐17. 10.1128/CMR.00088-17 30068738 PMC6148190

[40] Coelho, Luis P. , Renato Alves , Álvaro R. del Río , Pernille N. Myers , Carlos P. Cantalapiedra , Joaquín Giner‐Lamia , Thomas S. Schmidt , et al. 2022. “Towards the Biogeography of Prokaryotic Genes.” Nature 601: 252–256. 10.1038/s41586-021-04233-4 34912116 PMC7613196

[41] Nesme, Joseph , Sébastien Cécillon , Tom O. Delmont , Jean‐Michel Monier , Timothy M. Vogel , and Pascal Simonet . 2014. “Large‐Scale Metagenomic‐Based Study of Antibiotic Resistance in the Environment.” Current Biology 24: 1096–1100. 10.1016/j.cub.2014.03.036 24814145

[42] Chen, Daisy W. , and Nandita R. Garud . 2022. “Rapid Evolution and Strain Turnover in the Infant Gut Microbiome.” Genome Research 32: 1124–1136. 10.1101/gr.276306.121 35545448 PMC9248880

[43] Hu, Yongfei , Xi Yang , Jing Li , Na Lv , Fei Liu , Jun Wu , Ivan Y. C. Lin , et al. 2016. “The Bacterial Mobile Resistome Transfer Network Connecting the Animal and Human Microbiomes.” Applied and Environmental Microbiology 82: 6672–6681. 10.1128/AEM.01802-16 27613679 PMC5086561

[44] Groussin, Mathieu , Mathilde Poyet , Ainara Sistiaga , Sean M. Kearney , Katya Moniz , Mary Noel , Jeff Hooker , et al. 2021. “Elevated Rates of Horizontal Gene Transfer in the Industrialized Human Microbiome.” Cell 184: 2053–2067.e18. 10.1016/j.cell.2021.02.052 33794144

[45] Treiber, Michelle L. , Diana H. Taft , Ian Korf , David A. Mills , and Danielle G. Lemay . 2020. “Pre‐ and Post‐Sequencing Recommendations for Functional Annotation of Human Fecal Metagenomes.” BMC Bioinformatics 21: 74. 10.1186/s12859-020-3416-y 32093654 PMC7041091

[46] Truong, Duy T. , Eric A. Franzosa , Timothy L. Tickle , Matthias Scholz , George Weingart , Edoardo Pasolli , Adrian Tett , Curtis Huttenhower , and Nicola Segata . 2015. “MetaPhlAn2 for Enhanced Metagenomic Taxonomic Profiling.” Nature Methods 12: 902–903. 10.1038/nmeth.3589 26418763

[47] Li, Dinghua , Chi‐Man Liu , Ruibang Luo , Kunihiko Sadakane , and Tak Wah Lam . 2015. “MEGAHIT: An Ultra‐Fast Single‐Node Solution for Large and Complex Metagenomics Assembly Via Succinct De Bruijn Graph.” Bioinformatics 31: 1674–1676. 10.1093/bioinformatics/btv033 25609793

[48] Oren, Aharon , and George M. Garrity . 2021. “Valid Publication of the Names of Forty‐Two Phyla of Prokaryotes.” International Journal of Systematic and Evolutionary Microbiology 71: 005056. 10.1099/ijsem.0.005056 34694987

[49] Lakin, Steven M. , Chris Dean , Noelle R. Noyes , Adam Dettenwanger , Anne S. Ross , Enrique Doster , Pablo Rovira , et al. 2017. “MEGARes: An Antimicrobial Resistance Database for High Throughput Sequencing.” Nucleic Acids Research 45: D574–D580. 10.1093/nar/gkw1009 27899569 PMC5210519

[50] Li, Bing , Ying Yang , Liping Ma , Feng Ju , Feng Guo , James M. Tiedje , and Tong Zhang . 2015. “Metagenomic and Network Analysis Reveal Wide Distribution and Co‐Occurrence of Environmental Antibiotic Resistance Genes.” The ISME Journal 9: 2490–2502. 10.1038/ismej.2015.59 25918831 PMC4611512

[51] Taft, Diana H. , Jinxin Liu , Maria X. Maldonado‐Gomez , Samir Akre , M. Nazmul Huda , S. M. Ahmad , Charles B. Stephensen , and David A. Mills . 2018. “Bifidobacterial Dominance of the Gut in Early Life and Acquisition of Antimicrobial Resistance.” mSphere 3: e00441‐18. 10.1128/mSphere.00441-18 30258040 PMC6158511

[52] Oliver, Andrew , Zhengyao Xue , Yirui T. Villanueva , Blythe Durbin‐Johnson , Zeynep Alkan , Diana H. Taft , Jinxin Liu , et al. 2022. “Association of Diet and Antimicrobial Resistance in Healthy U.S. Adults.” mBio 13: e0010122. 10.1128/mbio.00101-22 35536006 PMC9239165

[53] Nayfach, Stephen , and Katherine S. Pollard . 2015. “Average Genome Size Estimation Improves Comparative Metagenomics and Sheds Light on the Functional Ecology of the Human Microbiome.” Genome Biology 16: 51. 10.1186/s13059-015-0611-7 25853934 PMC4389708

[54] Dröge, J. , I. Gregor , and A. C. Mchardy . 2015. “Taxator‐Tk: Precise Taxonomic Assignment of Metagenomes by Fast Approximation of Evolutionary Neighborhoods.” Bioinformatics 31: 817–824. 10.1093/bioinformatics/btu745 25388150 PMC4380030

[55] Menzel, Peter , Kim L. Ng , and Anders Krogh . 2016. “Fast and Sensitive Taxonomic Classification for Metagenomics With Kaiju.” Nature Communications 7: 11257. 10.1038/ncomms11257 PMC4833860 27071849

[56] Drula, Elodie , Marie‐Line Garron , Suzan Dogan , Vincent Lombard , Bernard Henrissat , and Nicolas Terrapon . 2022. “The Carbohydrate‐Active Enzyme Database: Functions and Literature.” Nucleic Acids Research 50: D571–D577. 10.1093/nar/gkab1045 34850161 PMC8728194

[57] Buchfink, Benjamin , Chao Xie , and Daniel H. Huson . 2015. “Fast and Sensitive Protein Alignment Using DIAMOND.” Nature Methods 12: 59–60. 10.1038/nmeth.3176 25402007

[58] Zeng, Shuqin , Dhrati Patangia , Alexandre Almeida , Zhemin Zhou , Dezhi Mu , R. Paul Ross , Catherine Stanton , and Shaopu Wang . 2022. “A Compendium of 32,277 Metagenome‐Assembled Genomes and Over 80 Million Genes From the Early‐Life Human Gut Microbiome.” Nature Communications 13: 5139. 10.1038/s41467-022-32805-z PMC9437082 36050292

[59] Clarke, K. Robert 1993. “Non‐Parametric Multivariate Analyses of Changes in Community Structure.” Australian Journal of Ecology 18: 117–143. 10.1111/j.1442-9993.1993.tb00438.x

[60] Nakagawa, Shinichi , and Holger Schielzeth . 2012. “A General and Simple Method for Obtaining R2 From Generalized Linear Mixed‐Effects Models.” Methods in Ecology and Evolution 4: 133–142. 10.1111/j.2041-210x.2012.00261.x

[61] Ziyatdinov, Andrey , Miquel Vázquez‐Santiago , Helena Brunel , Angel Martinez‐Perez , Hugues Aschard , and Jose M. Soria . 2018. “lme4qtl: Linear Mixed Models With Flexible Covariance Structure for Genetic Studies of Related Individuals.” BMC Bioinformatics 19: 68. 10.1186/s12859-018-2057-x 29486711 PMC5830078

[62] Difford, Gareth F. , Damian R. Plichta , Peter Løvendahl , Jan Lassen , Samantha J. Noel , Ole Højberg , André‐Denis G. Wright , et al. 2018. “Host Genetics and the Rumen Microbiome Jointly Associate With Methane Emissions in Dairy Cows.” PLoS Genetics 14: e1007580. 10.1371/journal.pgen.1007580 30312316 PMC6200390

[63] Xue, Mingyuan , Huizeng Sun , Xuehui Wu , Jianxin Liu , and Leluo Guan . 2020. “Multi‐Omics Reveals That the Rumen Microbiome and Its Metabolome Together With the Host Metabolome Contribute to Individualized Dairy Cow Performance.” Microbiome 8: 64. 10.1186/s40168-020-00819-8 32398126 PMC7218573

[64] Holmes, Ian , Keith Harris , and Christopher Quince . 2012. “Dirichlet Multinomial Mixtures: Generative Models for Microbial Metagenomics.” PLoS One 7: e30126. 10.1371/journal.pone.0030126 22319561 PMC3272020

[65] Bortolaia, Valeria , Rolf S. Kaas , Etienne Ruppe , Marilyn C. Roberts , Stefan Schwarz , Vincent Cattoir , Alain Philippon , et al. 2020. “ResFinder 4.0 for Predictions of Phenotypes From Genotypes.” Journal of Antimicrobial Chemotherapy 75: 3491–3500. 10.1093/jac/dkaa345 32780112 PMC7662176

