# Supplementary material for: Infant age inversely correlates with gut carriage of resistance genes, reflecting modifications in microbial carbohydrate metabolism during early life
Source: Imeta. 2024 Jan 31;3(2):e169. doi: 10.1002/imt2.169 (PMC11170968; doi:10.1002/imt2.169)
Supplement: Supplementary file 1 — Figure S1: The attributions (counts, lengths, and qualities) of raw and processed sequencing reads. Figure S2: Sankey diagram connecting resistance genes from month 0‐14 at the antimicrobial compound class level (left) to the predicted bacterial hosts at the genus (middle) and phylum level (right). Figure S3: The dynamics of resistome in Bifidobacterium and Escherichia genome. Figure S4: Clinical variables significantly associated with the inverse‐normal transformed absolute abundance of summed resistance. Figure S5: Forest floor main effect plots of random forest mapping structure of model predicting panel ratings of infant age on basis of MEGID. Figure S6: Heatmap of age‐associated MEGID and CAZy enzymes mapped in certain genus of metagenome‐assembled genomes (MAGs). Figure S7: Flow chart of systematic review and selection process. [file IMT2-3-e169-s001.docx]

**Supporting information to:**

**Infant age inversely correlates with gut carriage of resistance genes, reflecting modifications in microbial carbohydrate metabolism during early life**

**Running title: Infant resistome**

Xinming Xu^1,2,6,8#^, Qingying Feng^1,2,10#^, Tao Zhang^1,2#^, Yunlong Gao^1,2#^, Qu Cheng^9^, Wanqiu Zhang^1,2^, Qinglong Wu^12^, Ke Xu^13^, Yucan Li^14^, Nhu Nguyen^3^, Diana H. Taft^3^, David A. Mills^3,4^, Danielle G. Lemay^5^, Weiyun Zhu^1,2^, Shengyong Mao^1,2^, Anyun Zhang^11*^, Kelin Xu^7*^, Jinxin Liu^1,2*^

^1^Laboratory of Gastrointestinal Microbiology, College of Animal Science &Technology, Nanjing Agricultural University, Nanjing 210095, China

^2^Jiangsu Key Laboratory of Gastrointestinal Nutrition and Animal Health, National Center for International Research on Animal Gut Nutrition, Nanjing Agricultural University, Nanjing 210095, China

^3^Department of Food Science & Technology, University of California, Davis, One Shields Ave., Davis, CA 95616, USA

^4^Department of Viticulture and Enology, Robert Mondavi Institute for Wine and Food Science, University of California, Davis, One Shields Ave., Davis, CA 95616, USA

^5^USDA ARS Western Human Nutrition Research Center, 430 West Health Sciences Dr., Davis, CA 95616, USA

^6^Institutes of Biomedical Sciences, Fudan University, Shanghai 200030, China

^7^Department of Biostatistics, School of Public Health, Key Laboratory of Public Health Safety, NHC Key Laboratory of Health Technology Assessment, Fudan University, Shanghai 200030, China

^8^Department of Nutrition and Food Hygiene, School of Public Health, Institute of Nutrition, Fudan University, Shanghai 200030, China

^9^Department of Epidemiology and Biostatistics, School of Public Health, Tongji Medical College, Huazhong University of Science and Technology, Wuhan 430030, China

^10^Biological Engineering Division, Massachusetts Institute of Technology (MIT), Cambridge, MA 02139, USA.

^11^Animal Disease Prevention and Food Safety Key Laboratory of Sichuan Province, Key Laboratory of Bio-Resource and Eco-Environment of Ministry of Education, College of Life Sciences, Sichuan University, Chengdu 610010, China

^12^Department of Pathology and Immunology, Baylor College of Medicine, Houston, TX 77030, USA

^13^Department of Statistics, University of Chicago, 5747 South Ellis Avenue, Chicago, IL 60637

^14^Human Phenome Institute, Fudan University, Shanghai 200438, China

^#^ The authors contributed equally:

Author Xu, Author Feng, Author Zhang, Author Gao

^*^Correspondence: [jxnliu@njau.edu.cn](mailto:jxnliu@njau.edu.cn) (Author Liu), zhanganyu@scu.edu.cn (Author Zhang), [xukelin@fudan.edu.cn](mailto:xukelin@fudan.edu.cn) (Author Xu)


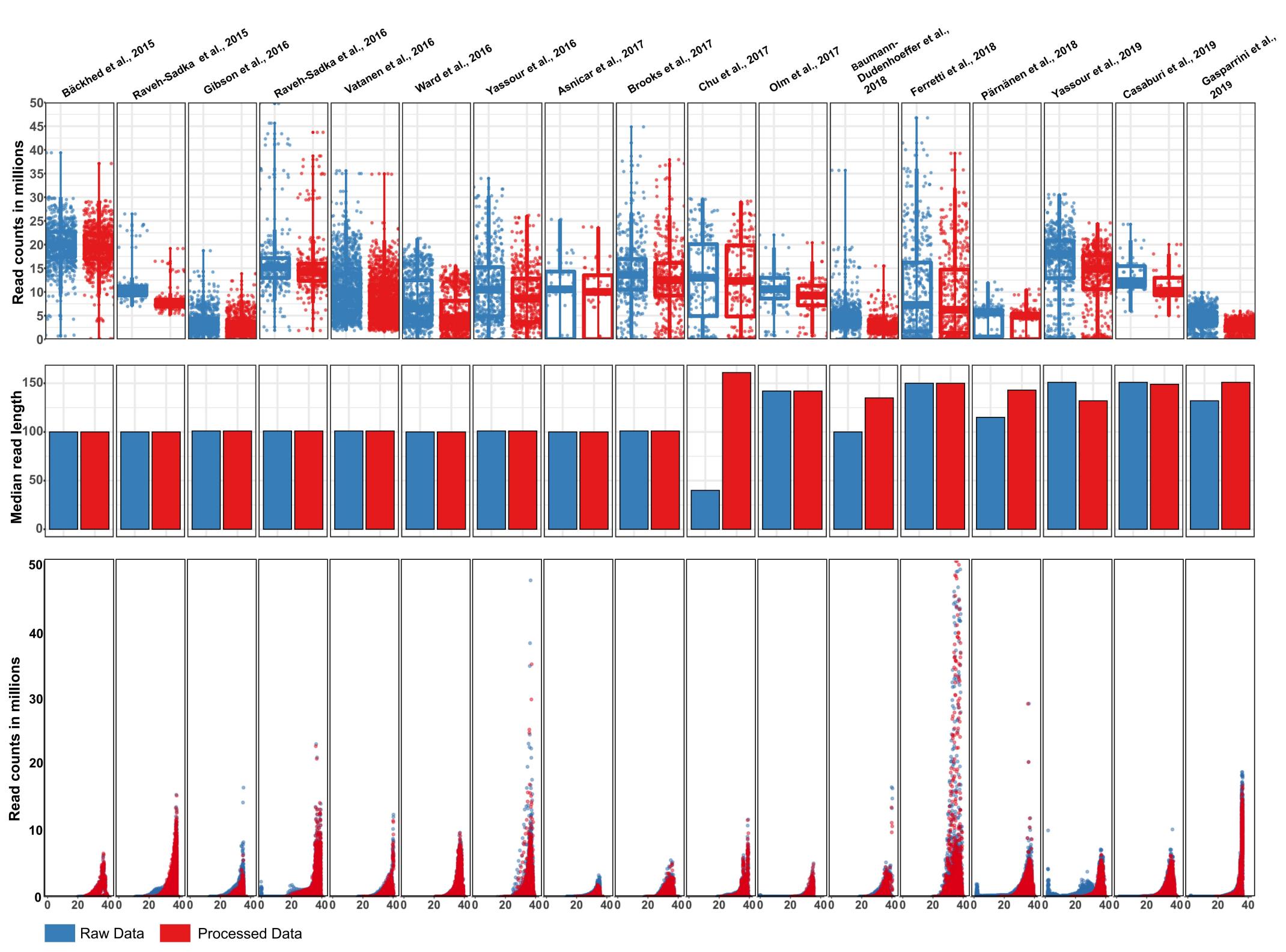


**Figure S1 The attributions (counts, lengths, and qualities) of raw and processed sequencing reads.** The first-row depicting the distribution of the read counts of raw data (Raw) and the clean data (Clean) within a study. Raw: raw data; Clean: clean data following quality control (after Trimmomatic and BMTagger); the second-row depicting descriptive information about the median read length range of each sample reads for each study included in our analysis. All reads in Brooks *et al*., 2017 are single-ended reads; the third-row depicting the distribution of the read quality of raw data and the clean data within a study. Blue dots refer to raw data, and red dots refer to clean data after quality control (after Trimmomatic and BMTagger). X axis refers to the read quality and Y axis refers to the number of read count. Color in blue refers to raw data, and red refers to processed data


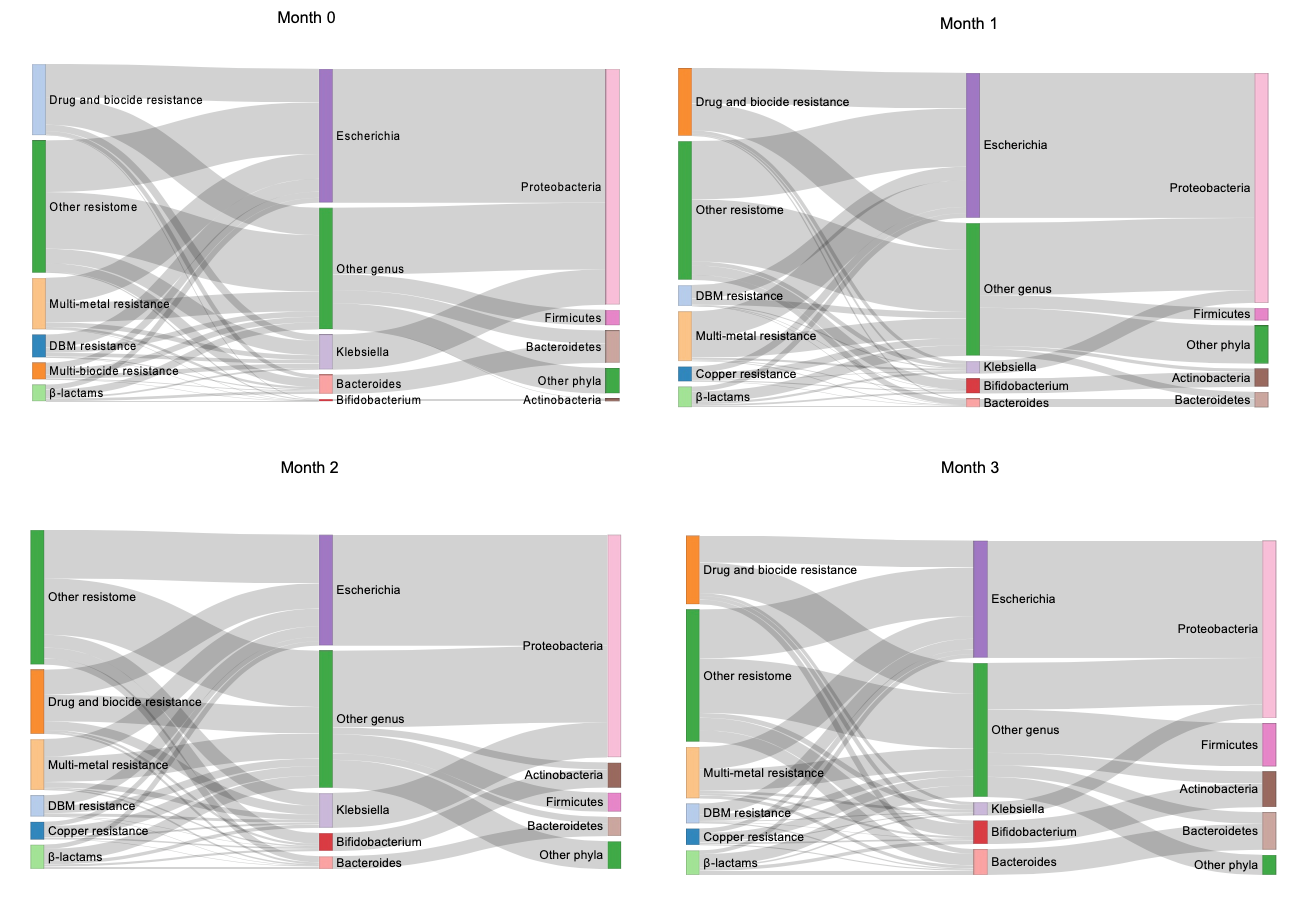


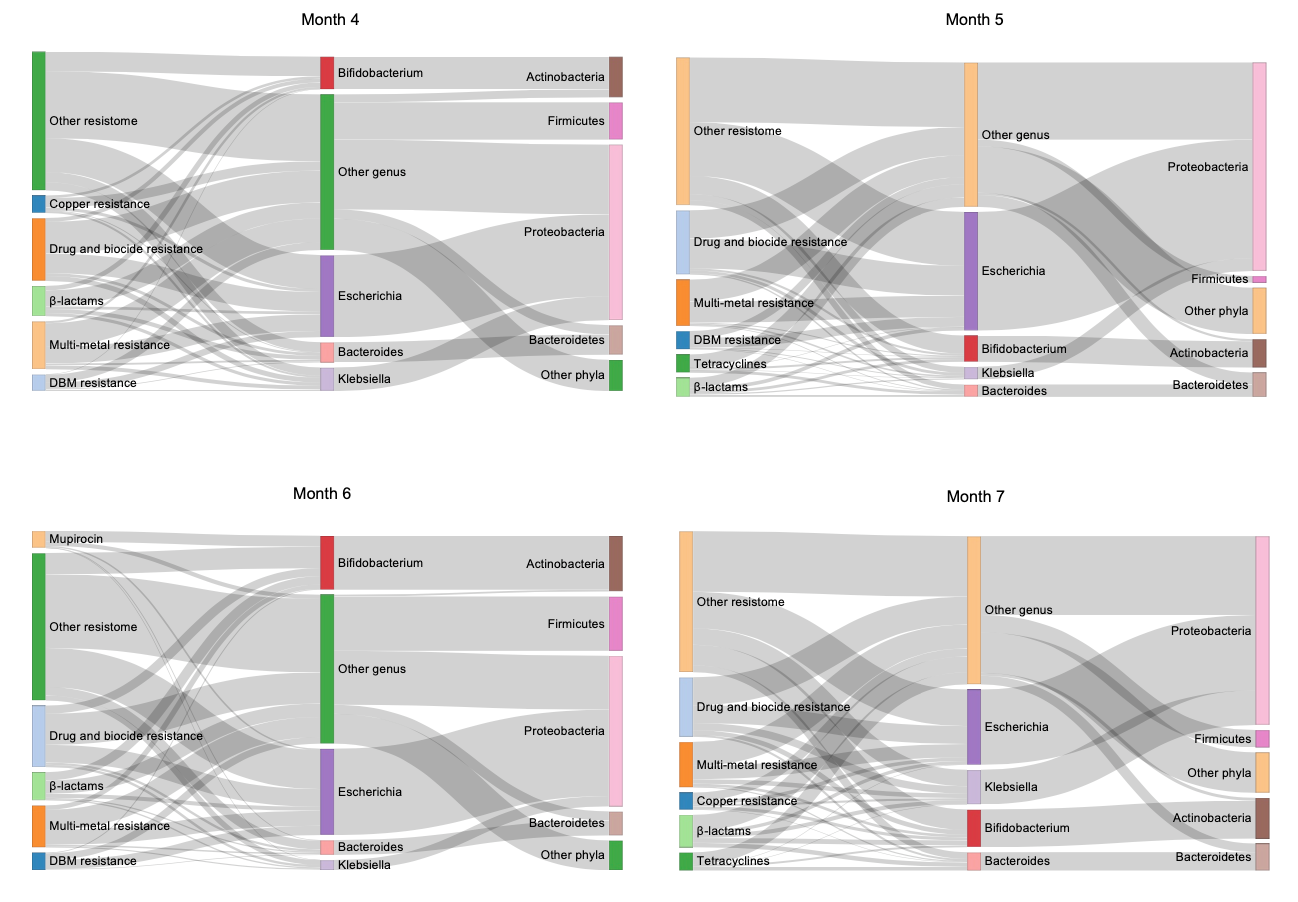


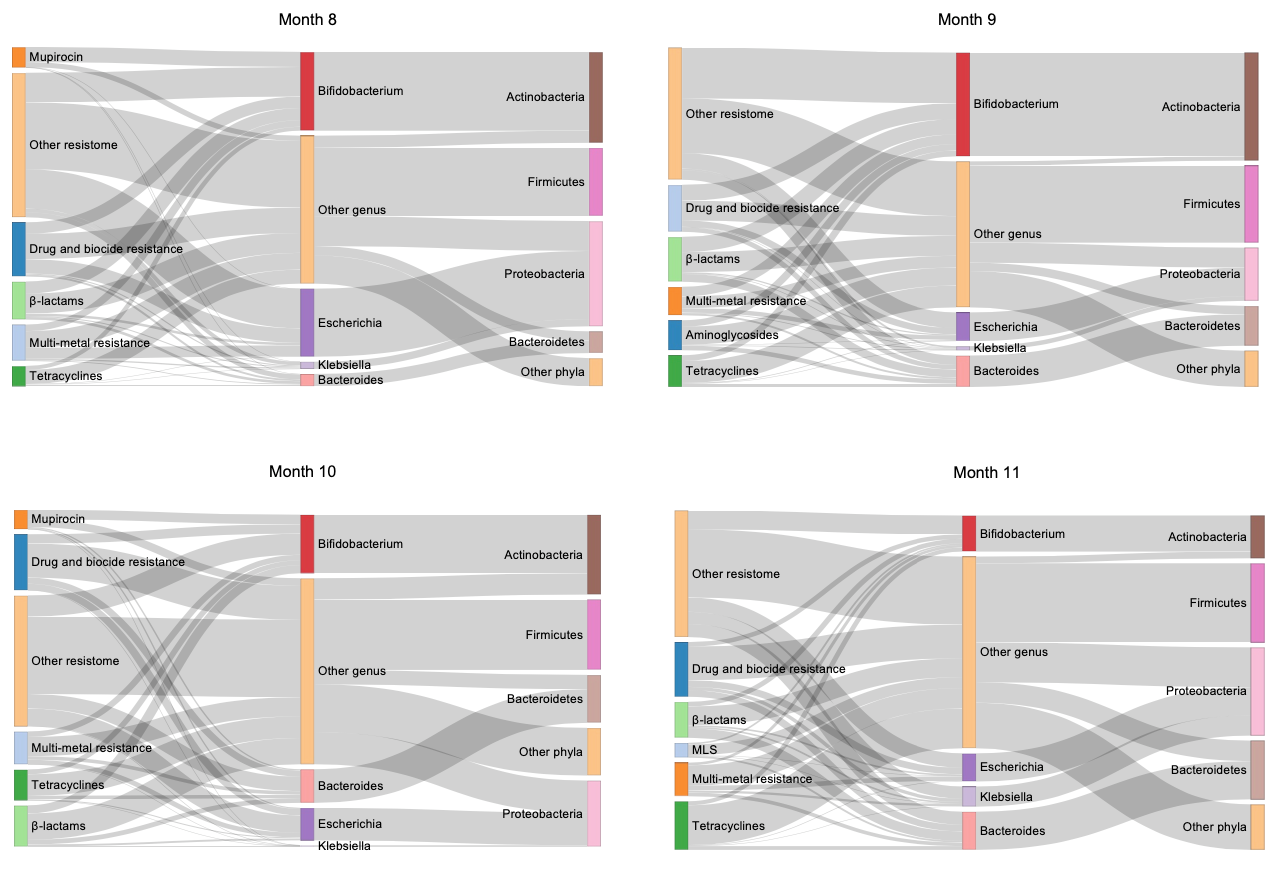


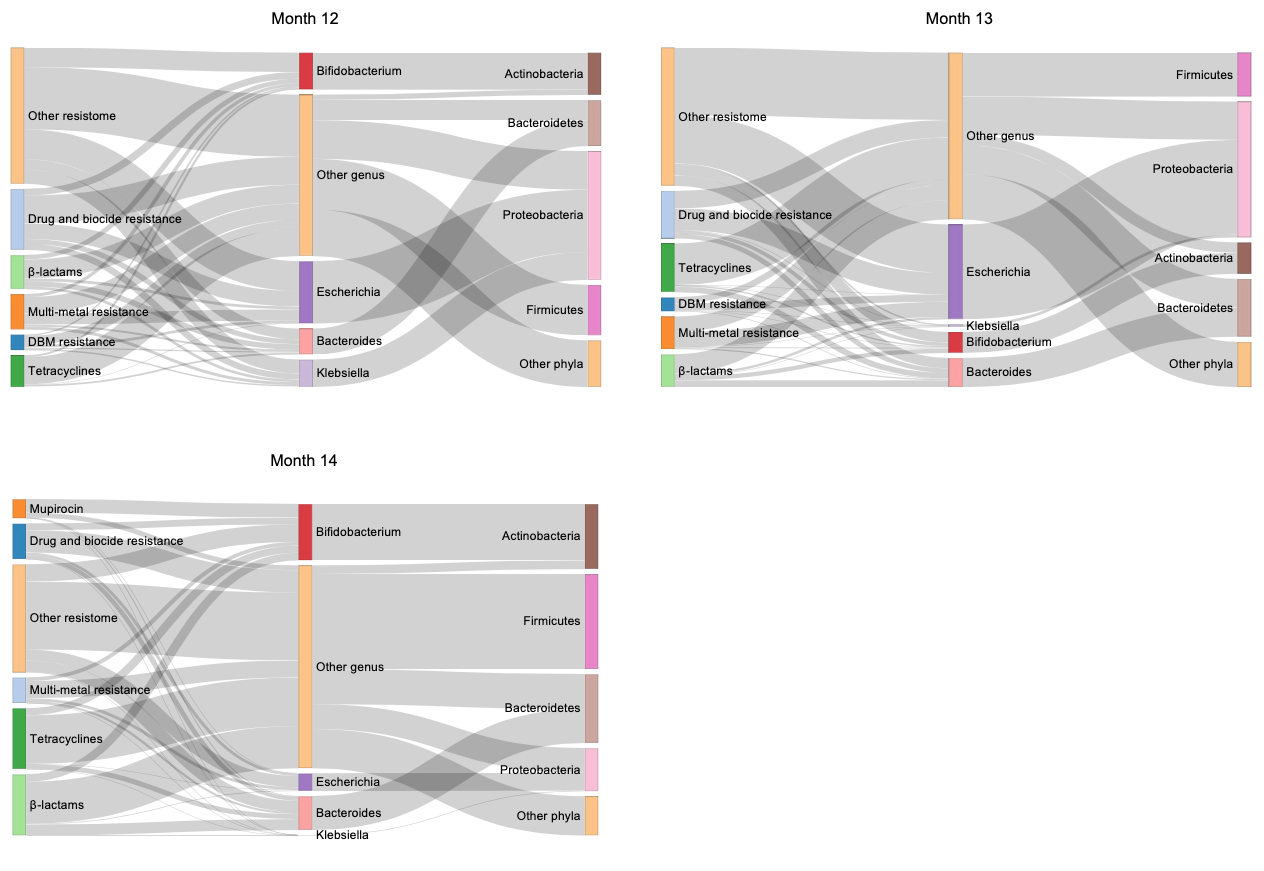


**Figure S2 Sankey diagram connecting resistance genes from month 0-14 at the antimicrobial compound class level (left) to the predicted bacterial hosts at the genus (middle) and phylum level (right).** For better visualization, phyla and genus were respectively limited within top 4 phyla and top 4 genus carrying the most resistance (seen in Figure 3B, C), otherwise will be aggregated to other phyla or other genus. The resistance is filtered by the core resistance and top5 core resistome in each month was shown, otherwise would be aggregated into other resistome.


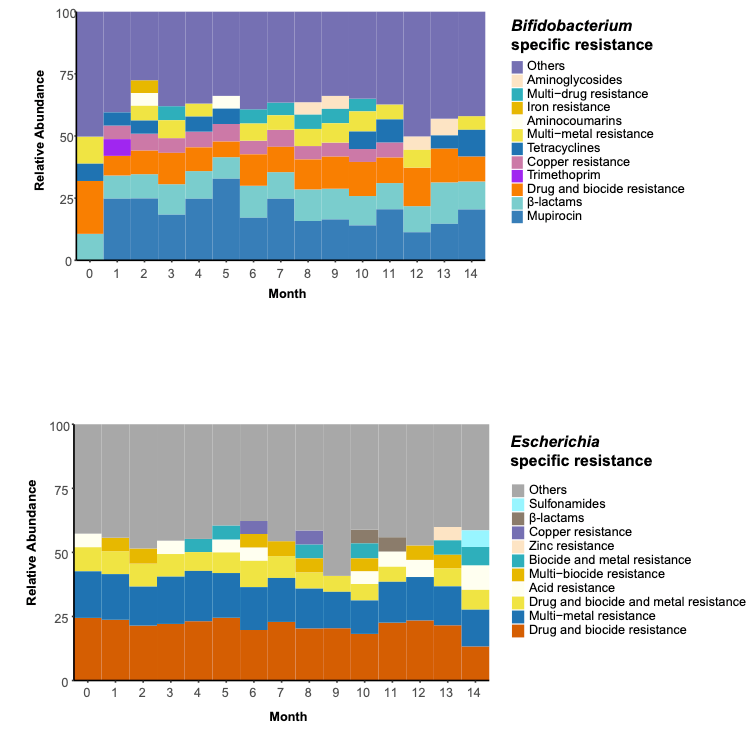


**Figure S3 The dynamics of resistome in *Bifidobacterium* and *Escherichia* genome.** Resistances are listed in the order (top-bottom: low-high).


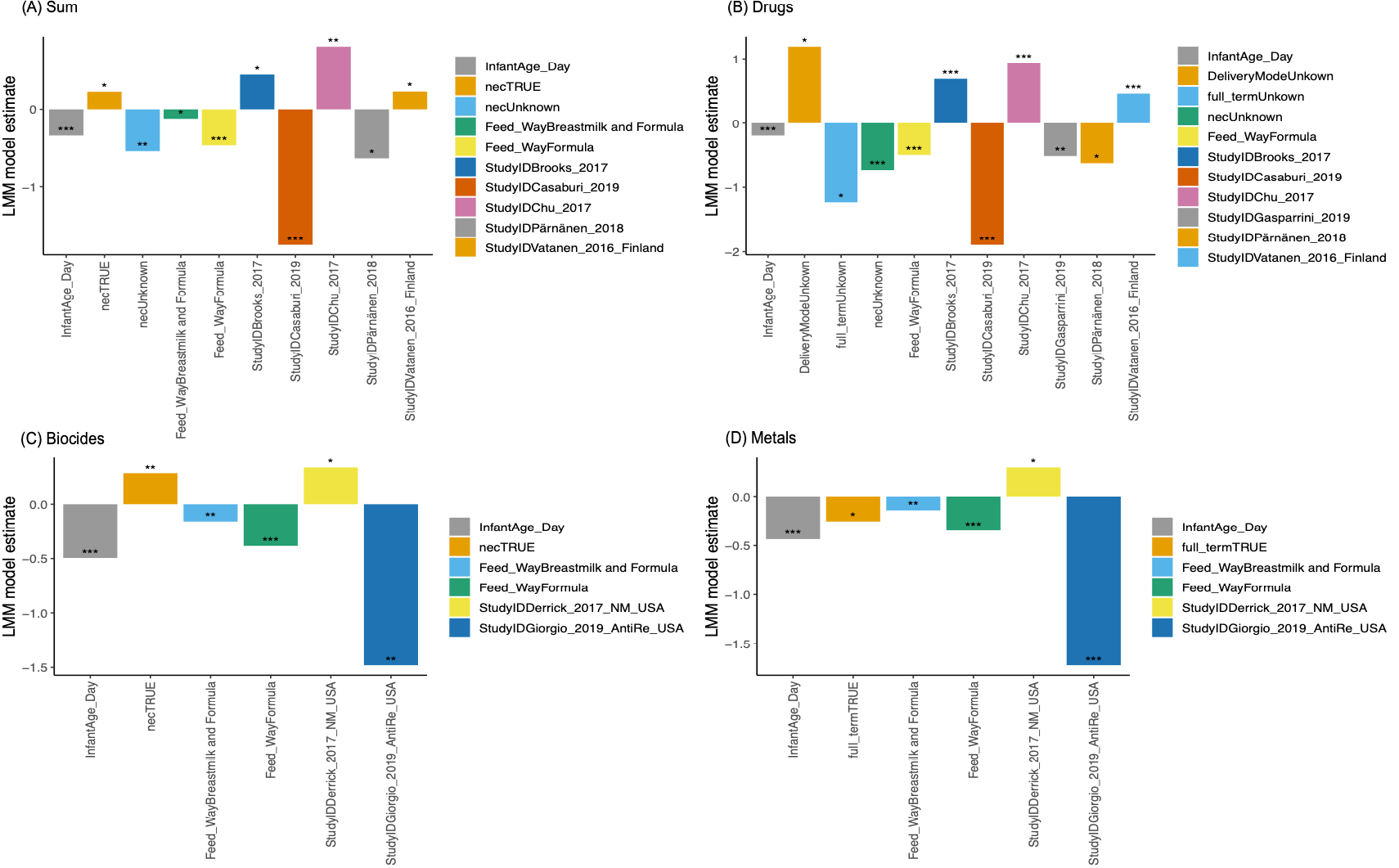


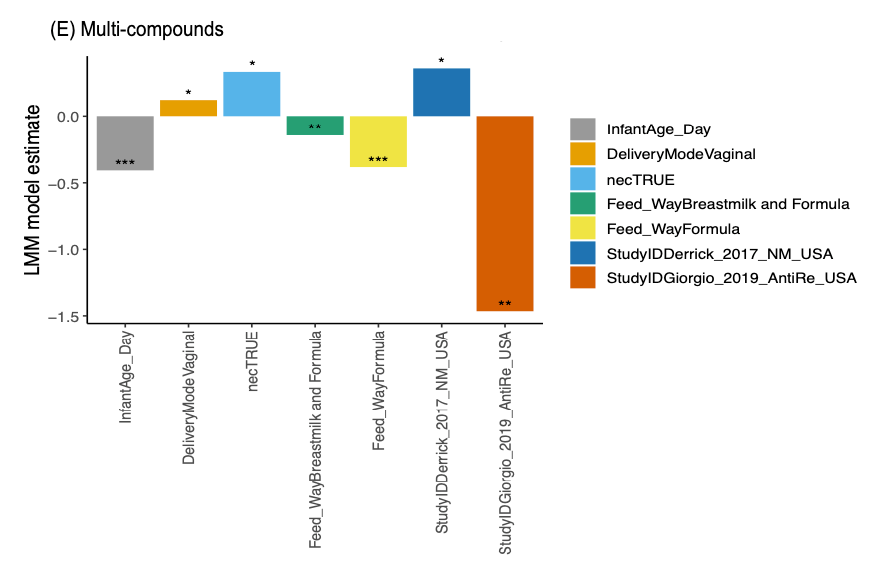


**Figure S4 Clinical variables significantly associated with the inverse-normal transformed absolute abundance of summed resistance.** (A) Sum. (B) Drugs. (C) Biocides. (D) Metals. (E) Multi-compounds.


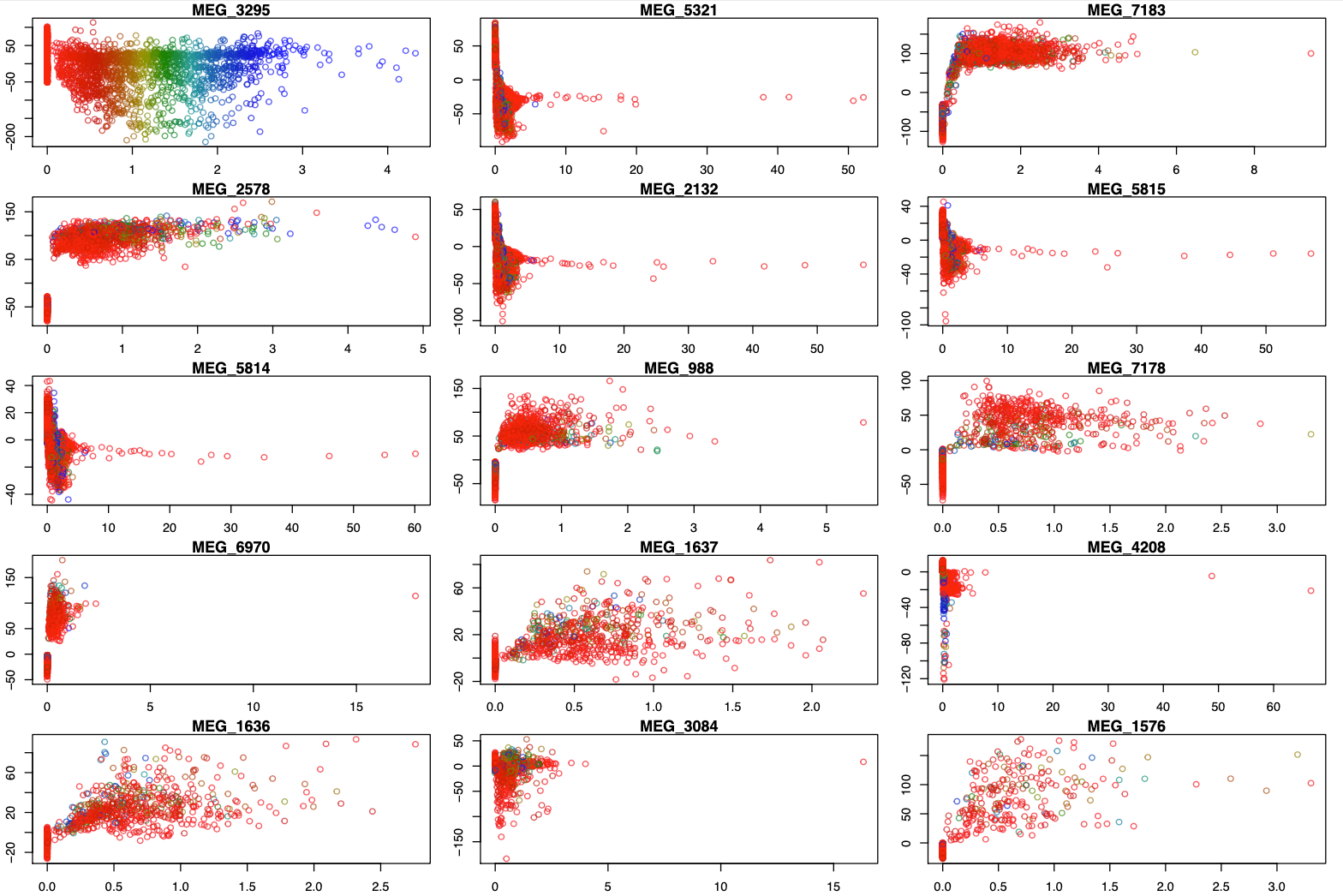


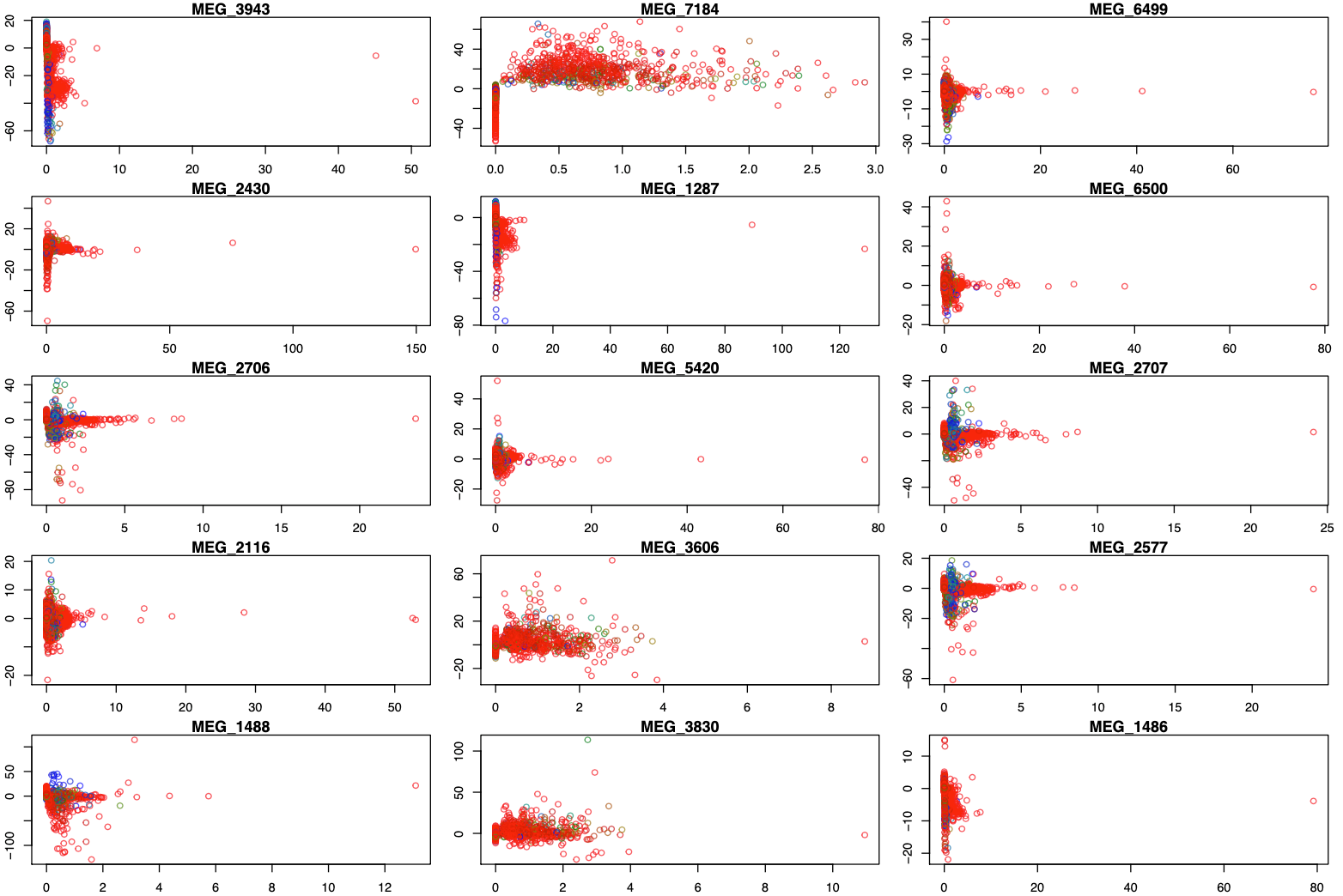


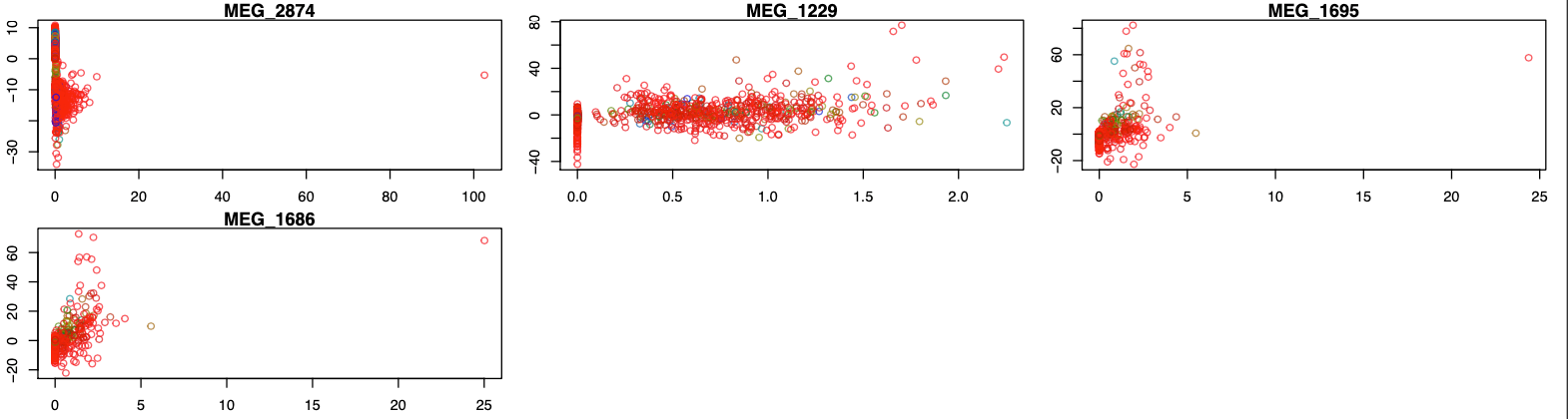


**Figure S5 Forest floor main effect plots of random forest mapping structure of model predicting panel ratings of infant age on basis of MEGID.** The plots are arranged according to variable importance. X-axis are the square roots of variable values - ARG absolute abundance for better visualization and Y-axis the change of predicted age due to resistance value. R^2^ quantifies the goodness-of-fit when visualizing the variable effect as on main effect. Color gradient color all samples by MEG 3295. Color gradient show how other variables interact with MEG3295.

**Figure S6 Heatmap of age-associated MEGID and CAZy enzymes mapped in certain genus of metagenome-assembled genomes (MAGs).** 110 age-associated MEGID (blue) and 83 age-associated CAZy enzymes (pink) were mapped in selected MAGs. A total of 8927 MAGs were obtained in four genera (802, 2069, 4560, 1496 in *Escherchia*, *Klebsiella*, *Bifidobacterium*, *Bacteroides*, respectively). The heatmap indicated the presence or absence of specific MEGID and CAZy enzymes, and the absence of a dot indicated that the specific MEGID and CAZy enzymes was not detected in specific MAG.


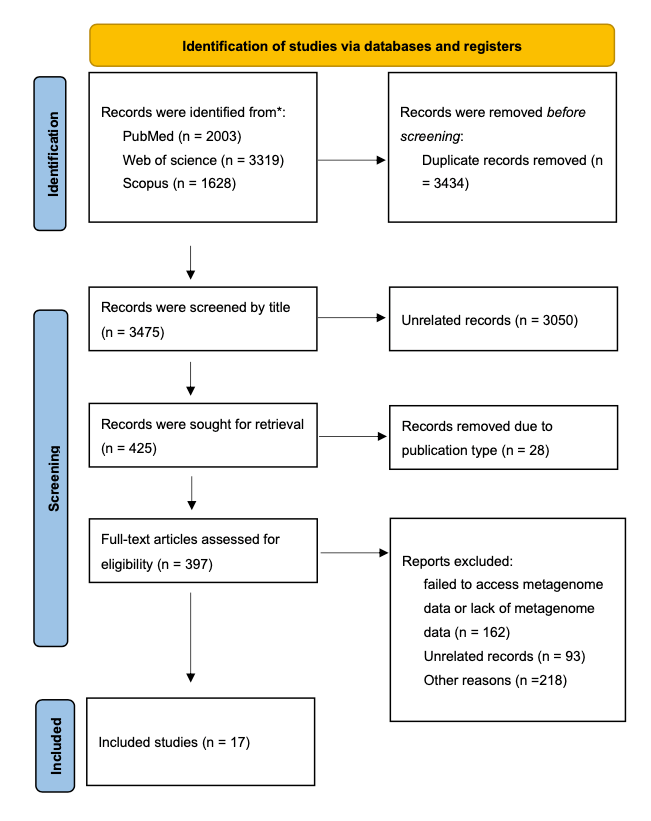


**Figure S7 Flow chart of systematic review and selection process.**
